# Five new 5,6-*seco*-tremulane sesquiterpenoids from the basidiomycete *Conocybe siliginea*

**DOI:** 10.1007/s13659-013-0003-1

**Published:** 2013-03-21

**Authors:** Xiao-Yan Yang, Tao Feng, Jian-Hai Ding, Xia Yin, Hua Guo, Zheng-Hui Li, Ji-Kai Liu

**Affiliations:** 13 State Key Laboratory of Phytochemistry and Plant Resources in West China, Kunming Institute of Botany, Chinese Academy of Sciences, Kunming, 650201 China; 23 University of Chinese Academy of Sciences, Beijing, 100049 China

**Keywords:** *Conocybe siliginea*, 5,6-*seco*-tremulane sesquiterpenoids

## Abstract

Five new 5,6-*seco*-tremulane sesquiterpenoids (**1–5**), as well as three known analogues (**6–8**), were isolated from the basidiomycete *Conocybe siliginea*. The structures of new compounds were elucidated by extensive spectroscopic methods. The known compounds were identified by comparing their spectroscopic data with those reported in the literature.

**Electronic Supplementary Material:**

Supplementary material is available for this article at 10.1007/s13659-013-0003-1 and is accessible for authorized users.

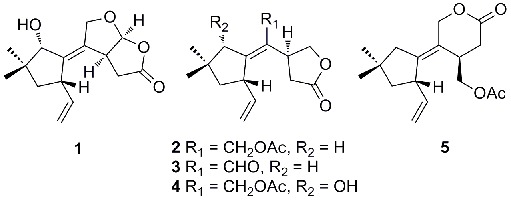

## Introduction

The genus *Conocybe* belongs to the order Agaricales and family Bolbitiaceae. Our previous study on the secondary metabolites of the *Conocybe siliginea* resulted in the isolation of a series of tremulane sesquiterpenoids.[1–3] As a part of our efforts to find the structurally diverse and biologically active secondary metabolites from higher fungi,[4–8] a further investigation on *C. siliginea* has led to the isolation of five new sesquiterpenoids, 11,12-epoxy-10*α*-hydroxy-5,6-*seco*-1,6(13)-tremuladien-5,12-olide (**1**), 11-acetoxy-5,6-*seco*-1,6(13)-tremuladien-5,12-olide (**2**), 11-aldehyde-5,6-*seco*-1,6(13)-tremuladien-5,12-olide (**3**), 11-acetoxy-10*α*-hydroxy-5,6-*seco*-1,6(13)-tremuladien-5,12-olide (**4**), and 12-acetoxy-5,6-*seco*-1,6(13)-tremuladien-5,11-olide (**5**). The new compounds were elucidated by means of spectroscopic methods, which represented 5,6-*seco*-tremulane sesquiterpenoids. By comparison with spectroscopic data reported in the literature, three known compounds were identified as conocenolide A (**6**),[2] conocenolide B (**7**),[2] and 10*β*,11-dihydroxy-5,6-*seco*-1,6(13)-tremuladien-5,12-olide (**8**).[1] So far, 5,6-*seco*-tremulane sesquiterpenoids have been found limited to basidiomycete *C. siliginea*[1–3] and *Irepex lacteus*,[9] and only dermatolactone showed cytotoxic activity and weak antimicrobial activity.[9] This paper describes the isolation and structure elucidation of five new 5,6-*seco*-tremulane sesquiterpenoids.

**1**

**2** $R_1 = CH_2OAc$, $R_2 = H$
**3** $R_1 = CHO$, $R_2 = H$
**4** $R_1 = CH_2OAc$, $R_2 = OH$

**5** R = OAc
**7** R = OH

**6** R = H
**8** R = OH

## Results and Discussion

Compound **1** was obtained as colorless oil. Its molecular formula $C_{15}H_{20}O_4$ was established by the HREIMS at *m/z* 264.1363 $[M]^+$ (calcd. for 264.1362), indicating six degrees of unsaturation. The IR spectrum indicated the presence of hydroxyl (3434 $cm^{-1}$) and carbonyl groups (1779 $cm^{-1}$). The $^1H$ and $^{13}C$ NMR data (Table 1) revealed the existence of two methyls, four methylenes (one oxygenated and one olefinic), five methines (two oxygenated and one olefinic), and four quaternary carbons (two olefinic and one carbonyl). Comparison of $^1H$ and $^{13}C$ NMR data of **1** (Table 1) with those of 10*β*,11-dihydroxy-5,6-*seco*-1,6(13)-tremuladien-5,12-olide (**8**)[1] showed that they are similar in structure, except for the absence of an oxymethylene ($\delta_C$ 70.8, C-12) instead of the appearance of an acetal carbon ($\delta_H$ 6.10, $\delta_C$ 107.9 ) in **1**, as

*To whom correspondence should be addressed. E-mail: jkliu@mail.kib.ac.cn

Springer

**Table 1. $^1$H and $^{13}$C NMR data of 1 and 2 ($CDCl_3$, $\delta$ in ppm and $J$ in Hz)**

| | 1 | | 2 | |
|---|---|---|---|---|
| position | $\delta_H$ ($J$ in Hz) | $\delta_C$ | $\delta_H$ ($J$ in Hz) | $\delta_C$ |
| 1 | | 140.4, C | | 151.1, C |
| 2 | | 137.3, C | | 124.7, C |
| 3 | 3.63, m | 40.8, CH | 3.82, m | 36.8, CH |
| 4a | 2.90, dd (19.0, 11.0, Ha); 2.54, dd (19.0, 4.3, Hb) | 36.2, $CH_2$ | 2.57, dd (17.6, 9.0, Ha); 2.41, dd (17.6, 9.0, Hb) | 32.9, $CH_2$ |
| 5 | | 175.4, C | | 177.0, C |
| 6 | 5.74, m | 142.9, CH | 5.70, m | 141.7, CH |
| 7 | 3.27, m | 46.1, CH | 3.36, m | 46.2, CH |
| 8 | 1.70, m | 44.5, $CH_2$ | 1.81, m (Ha); 1.38, dd (12.8, 8.6, Hb) | 48.2, $CH_2$ |
| 9 | | 41.9, C | | 37.8, C |
| 10 | 3.85, s | 81.7, CH | 2.28 dd (15.6, 2.0, Ha); 2.16 br. d (15.6, Hb) | 46.4, $CH_2$ |
| 11 | 4.61, d (12.8, Ha); 4.55, d (12.8, Hb) | 68.7, $CH_2$ | 4.67, d (12.6, Ha); 4.59, d (12.6, Hb) | 62.2, $CH_2$ |
| 12 | 6.10, d (5.6) | 107.9, CH | 4.35, dd (8.8, 8.8, Ha); 4.09, overlap (Hb) | 70.8, $CH_2$ |
| 13 | 5.07, dd (17.0, 1.4, Ha); 4.99, dd (9.8, 1.4, Hb) | 114.8, $CH_2$ | 5.00, overlap | 113.8, $CH_2$ |
| 14 | 1.05, s | 21.9, $CH_3$ | 1.08 s | 28.5, $CH_3$ |
| 15 | 0.85, s | 25.8, $CH_3$ | 0.87 s | 27.1, $CH_3$ |
| $CH_3$*C*OO- | | | | 170.8, C |
| *$CH_3$*COO- | | | 2.04 s | 20.9, $CH_3$ |

deduced by the HMBC correlations from H-3 and C-12 and from H-12 to C-5 (Figure 1). In addition, the HMBC correlation from H-11 to C-12 established an epoxy moiety between C-11 and C-12 (Figure 1), which was in agreement with the degrees of unsaturation (Figure 1). According to the biogenetic origin, compound **1** may be biosynthesized via Baeyer-Villiger oxidation, elimination, oxidation, and esterification of conoceol B.[2] Therefore, the relative configuration of **1** should be consistent with that of conoceol B. In the ROESY spectrum, the observed correlations of H-3/H-7, H-3/H-12, Me-15/H-10, and Me-15/H-7 further identified the relative configuration as depicted (Figure 1). Consequently, compound **1** was elucidated as 11,12-epoxy-10*α*-hydroxy-5,6-*seco*-1,6(13)-tremuladien-5,12-olide.

**Figure 1.** Key 2D NMR correlations of **1**

Compound **2** had the molecular formula $C_{17}H_{24}O_4$ as established by the HRESIMS at *m/z* 315.1568 $[M + Na]^+$ (calcd. for 315.1572). The $^1$H and $^{13}$C NMR data (Table 1) were closely related to those of conocenolide A (**6**),[2] except for an additional acetoxy group [$\delta_H$ 2.04 (3H, s); $\delta_C$ 20.9 (q) and 170.8 (s)] in **2**. Compound **2** could be readily identified as the 11-acetoxy of conocenolide A by the downfield chemical shift of H-11 at $\delta_H$ 4.67 (1H, d, $J$ = 12.6 Hz) and 4.59 (1H, d, $J$ = 12.6 Hz), as well as the HMBC correlation from H-11 to $\delta_C$ 170.8 (s, $CH_3CO_2$-). Further analysis of 2D NMR data (HSQC, HMBC, $^1$H-$^1$H COSY, ROESY) suggested that the other parts of **1** were the same to those of **6**.[2] Therefore, compound **2** was determined to be 11-acetoxy-5,6-*seco*-1,6(13)-tremuladien-5,12-olide, as shown.

Compound **3** was a colorless oil and gave a molecular formula $C_{15}H_{20}O_3$, as assigned by HRESIMS at *m/z* 271.1316 $[M + Na]^+$ (calcd. for 271.1310). Detailed comparison of $^1$H and $^{13}$C NMR data (Table 2) of **3** with those of **6**[2] showed that they are similar in structure. The key difference was a CHO group ($\delta_H$ 9.94, $\delta_C$ 191.7) in **3** rather than an oxymethylene in **6**. The aldehyde was assigned to C-11 on the basis of HMBC correlations from $\delta_H$ 9.94 (1H, s, H-11) to $\delta_C$ 132.9 (s, C-2) and 34.5 (d, C-3). Analysis of 2D NMR data established the structure of **3** to be 11-aldehyde-5,6-*seco*-1,6(13)-tremuladien-5,12-olide, as shown.

Compound **4** possessed a molecular formula $C_{17}H_{24}O_5$, as deduced from HRESIMS at *m/z* 331.1532 $[M + Na]^+$ (calcd. for 331.1521). Comparison of $^1$H and $^{13}$C NMR data (Table 2) of **4** with those of **2** indicated that **4** possessed an additional hydroxyl group at C-10, which was determined by the HMBC correlations from $\delta_H$ 4.17 (1H, s, H-10) to C-7, C-8, C-1, C-2, and C-15, while the *α* orientation of OH-10 was deduced from ROESY correlations of H-10/Me-15 and Me-15/H-7. Hence, compound **4** was determined to be 11-acetoxy-10*α*-hydroxy-5,6-*seco*-1,6(13)-tremuladien-5,12-olide, as shown.

Compound **5** was obtained as a colorless oil with a molecular formula of $C_{17}H_{24}O_4$ established by the HRESIMS at *m/z* 315.1577 $[M + Na]^+$ (calcd. for 315.1572). The IR spectrum showed absorption for a carbonyl group (1742 $cm^{-1}$) and C=C double bond (1637 $cm^{-1}$). The $^1$H and $^{13}$C NMR data (Table 2) revealed the existence of three methyls, six methylenes (two oxygenated and one olefinic), three methines (one olefinic), and five quaternary carbons (two olefinic and two carbonyl). Detailed comparison of 1D and 2D NMR (HSQC, HMBC, ROESY) data of **5** with those of conocenolide B (**7**)[2] showed that they were similar in structure, except for an acetoxy substituent at C-12 in **5**, as indicated by the HMBC correlations from H-12 to $\delta_C$ 170.7 (s, $CH_3$*C*O-). Therefore, compound **5** was determined to be 12-acetoxy-5,6-*seco*-

**Table 2.** $^{1}$**H and** $^{13}$**C NMR data of 3–5 (CDCl$_3$,** $\delta$ **in ppm and** $J$ **in Hz)**

| | **3** | | **4** | | **5** | |
|---|---|---|---|---|---|---|
| position | $\delta_H$ | $\delta_C$ | $\delta_H$ | $\delta_C$ | $\delta_H$ | $\delta_C$ |
| 1 | | 172.5, C | | 152.5, C | | 144.3, C |
| 2 | | 132.9, C | | 129.2, C | | 122.5, C |
| 3 | 3.66, m | 34.5, CH | 3.79, m | 36.5, CH | 3.47, m | 32.4, CH |
| 4a | 2.78, dd (17.5, 9.5) | 31.7, CH$_2$ | 2.66, dd (17.9, 9.5) | 33.7, CH$_2$ | 2.61, m | 32.5, CH$_2$ |
| 4b | 2.46, overlap | | 2.34, dd (17.9, 7.2) | | | |
| 5 | | 177.2, C | | 176.8, C | | 172.1 C |
| 6 | 5.73, m | 139.6, CH | 5.76, m | 142.5, CH | 5.60, m | 139.6, CH |
| 7 | 3.62, m | 48.2, CH | 3.29, m | 45.1, CH | 3.44, m | 46.3, CH |
| 8a | 1.91, m | 46.7, CH$_2$ | 1.72, m | 44.0, CH$_2$ | 1.79, dd (12.7, 8.2) | 48.3, CH$_2$ |
| 8b | 1.44, dd (12.8, 8.6) | | | | 1.37, dd (12.7, 9.2) | |
| 9 | | 38.9, C | | 40.2, C | | 37.8, C |
| 10a | 2.92, dd (16.3, 1.6) | 45.4, CH$_2$ | 4.17, s | 79.5, CH | 2.10, m | 46.2, CH$_2$ |
| 10b | 2.48, overlap | | | | | |
| 11a | 9.94, s | 191.7, C | 5.11, d (12.8) | 62.3, CH$_2$ | 4.80, d (13.7) | 68.9, CH$_2$ |
| 11b | | | 4.61, d (12.8) | | 4.69, d (13.7) | |
| 12a | 4.35, t (9.0) | 70.3, CH$_2$ | 4.39, t (9.0) | 70.3, CH$_2$ | 4.15, dd (11.2, 4.7) | 64.4, CH$_2$ |
| 12b | 4.28, t (9.0) | | 4.16, t (9.0) | | 3.97, dd (11.2, 8.0) | |
| 13 | 5.07, overlap | 115.4, CH$_2$ | 5.00, m | 114.0, CH$_2$ | 5.08, d (17.2) | 114.7 CH$_2$ |
| | | | | | 5.01, d (10.0) | |
| 14 | 1.18, s | 28.3, CH$_3$ | 1.09, s | 21.8, CH$_3$ | 1.09, s | 28.4, CH$_3$ |
| 15 | 0.94, s | 27.0, CH$_3$ | 0.78, s | 25.7, CH$_3$ | 0.90, s | 27.2, CH$_3$ |
| CH$_3$*C*OO- | | | | 171.3, C | | 170.7 C |
| *CH$_3$*COO- | | | 2.07 s | 21.1, CH$_3$ | 2.05, s | 20.7, CH$_3$ |

1,6(13)-tremuladien-5,11-olide, as shown.

## Experimental Section

**General Experimental Procedures.** Optical rotations were measured on a Horiba SEPA-300 polarimeter. UV spectra were obtained using a Shimadzu UV-2401A spectrometer. IR spectra were obtained on a Bruker Tensor 27 spectrometer with KBr pellets. 1D and 2D NMR experiments were performed on a Bruker AM-400, DRX-500 or AVANCE III-600 spectrometer with TMS as the internal standard. Chemical shifts ($\delta$) were expressed in ppm with reference to the solvent signals. Mass spectra (MS) were recorded on a VG Auto Spec-3000 or an APIQSTAR time-of-flight spectrometer. Column chromatography (CC) was performed on silica gel (200–300 mesh; Qingdao Marine Chemical Ltd., China), RP-18 gel (40–75 $\mu$m, Fuji Silysia Chemical Ltd., Japan), and Sephadex LH-20 (Amersham Biosciences, Sweden). Fractions were monitored by TLC (GF$_{254}$, Qingdao Marine Chemical Ltd., China), and spots were visualized by spraying with 10% $H_2SO_4$ in ethanol.

**Fungal Material and Cultivation Conditions.** *C. siliginea* was isolated from the tissue culture of its fruiting bodies collected from a moist woodland (dominated by pines) of the Linglang county in Yunnan Province, China, in July 2003, and authenticated by Prof. Mu Zang, Kunming Institute of Botany, Chinese Academy of Sciences (CAS). A voucher specimen (KIB03071801) was deposited in the Herbarium of Kunming Institute of Botany, CAS. Culture medium: glucose 5%, peptone 0.15%, yeast powder 0.5%, $KH_2PO_4$ 0.05% and $MgSO_4$ 0.05%. Fermentation was carried out on a shaker at 250 r/min for 20 days.

**Extraction and Isolation.** The culture broth of *C. siliginea* (80 L) was filtered, and the filtrate was extracted four times with EtOAc. The organic layer was concentrated under reduced pressure to give an oily residue (40 g) that was subjected to column chromatography over silica gel (200–300 mesh) eluting with $CHCl_3$/MeOH (from 100:0 to 0:100) to afford fractions A–E. Fraction A was separated further by CC over RP-18, eluting with $H_2O$/MeOH (from 1:0 to 0:1) to give fractions $B_1$–$B_4$. Fraction $B_4$ was purified by repeated CC over silica gel (petroleum ether/EtOAc, 10:1) and then applied to Sephadex LH-20 ($Me_2CO$) to yield **2** (40.0 mg), **6** (25.2 mg), **7** (12.1 mg), Fraction $B_3$ was eluting with petroleum ether/EtOAc to give fractions $B_{3a}$–$B_{3g}$. Fraction $B_{3b}$ was purified by CC over silica gel (petroleum ether/EtOAc, 5:1) to yield **1** (48.0 mg), Fraction $B_{3e}$ by repeated silica gel CC (petroleum ether/EtOAc) and Sephadex LH-20 ($Me_2CO$) to yield **3** (4.2 mg), **4** (6.1 mg), **5** (3.5 mg) and **8** (9.5 mg).

**11,12-Epoxy-10*α*-hydroxy-5,6-*seco*-1,6(13)-tremuladien-5,12-olide (1):** colorless oil; $[\alpha]^{18}_{D}$ – 121.6 (*c* 0.40, MeOH); UV (MeOH) $\lambda_{max}$ (log $\varepsilon$) 220 (3.54), 204 (3.45) nm; IR (KBr) $\nu_{max}$ 3434, 2957, 2870, 1779, 1638, 1467, 1365, 1093, 1037, 984, 917cm$^{-1}$; $^{1}$H NMR (CDCl$_3$, 400 MHz) and $^{13}$C NMR (CDCl$_3$, 100 MHz) data, see Table 1; ESIMS (positive) *m/z* 287 [M + Na]$^+$; HREIMS (positive) *m/z* 264.1363 [M]$^+$ (calcd. for $C_{15}H_{20}O_4$, 264.1362).

**11-Acetoxy-5,6-*seco*-1,6(13)-tremuladien-5,12-olide (2):** colorless oil; $[\alpha]_D^{18}$ – 41.7 (*c* 0.40, MeOH); UV (MeOH) $\lambda_{max}$ (log $\varepsilon$) 218 (3.57) nm; IR (KBr) $\nu_{max}$ 2954, 2868, 1781, 1739, 1635, 1464, 1382, 1366, 1236, 1173, 1023, 916 $cm^{-1}$; $^1H$ NMR ($CDCl_3$, 400 MHz) and $^{13}C$ NMR ($CDCl_3$, 100 MHz) data, see Table 1; ESIMS (positive) *m/z* 315 $[M + Na]^+$; HRESIMS (positive) *m/z* 315.1568 $[M + Na]^+$ (calcd. for $C_{17}H_{24}O_4Na$, 315.1572).

**11-Aldehyde-5,6-*seco*-1,6(13)-tremuladien-5,12-olide (3):** colorless oil; $[\alpha]_D^{19}$ – 11.7 (*c* 0.29, MeOH); UV (MeOH) $\lambda_{max}$ (log $\varepsilon$) 249 (2.94), 201 (2.71) nm; IR (KBr) $\nu_{max}$ 2955, 2927, 2868, 1775, 1662, 1637, 1463, 1418, 1386, 1177, 1027 $cm^{-1}$; $^1H$ NMR ($CDCl_3$, 400 MHz) and $^{13}C$ NMR ($CDCl_3$, 100 MHz) data, see Table 2; ESIMS (positive) *m/z* 271 $[M + Na]^+$; HRESIMS (positive) *m/z* 271.1316 $[M + Na]^+$ (calcd. for $C_{15}H_{20}O_3Na$, 271.1310).

**11-Acetoxy-10*α*-hydroxy-5,6-*seco*-1,6(13)-tremuladien-5, 12-olide (4):** colorless oil; $[\alpha]_D^{19}$ – 26.6 (*c* 0.30, MeOH); UV (MeOH) $\lambda_{max}$ (log $\varepsilon$) 204 (3.04) nm; IR (KBr) $\nu_{max}$ 3460, 2957, 2935, 2871, 1778, 1740, 1636, 1467, 1418, 1380, 1237, 1176, 1023, 913$cm^{-1}$; $^1H$ NMR ($CDCl_3$, 400 MHz) and $^{13}C$ NMR ($CDCl_3$, 100 MHz) data, see Table 2; ESIMS (positive) *m/z* 331 $[M + Na]^+$; HRESIMS (positive) *m/z* 331.1532 $[M + Na]^+$ (calcd for $C_{17}H_{24}O_5Na$, 331.1521).

**12-Acetoxy-5,6-*seco*-1,6(13)-tremuladien-5,11-olide (5):** colorless oil; $[\alpha]_D^{20}$ + 10.9 (*c* 0.23, MeOH); UV (MeOH) $\lambda_{max}$ ( log $\varepsilon$) 205 (3.00) nm; IR (KBr) $\nu_{max}$ 2956, 2934, 2870, 1742, 1637, 1463, 1432, 1385, 1367, 1241, 1157, 1038, 918$cm^{-1}$; $^1H$ NMR ($CDCl_3$, 400 MHz) and $^{13}C$ NMR ($CDCl_3$, 100 MHz) data, see Table 2; ESIMS (positive) *m/z* 315 $[M + Na]^+$; HRESIMS (positive) *m/z* 315.1577 $[M + Na]^+$ (calcd. for $C_{17}H_{24}O_4Na$, 315.1572).

## Electronic Supplementary Material

Supplementary material is available in the online version of this article at http://dx.doi.org/ 10.1007/s13659-013-0003-1 and is accessible for authorized users.

## Acknowledgments

This project was supported by the National Basic Research Program of China (973 Program, 2009CB522300), the National Natural Sciences Foundation of China (U1132607), and Youth Innovation Promotion Association CAS.

**Open Access** This article is distributed under the terms of the Creative Commons Attribution License which permits any use, distribution, and reproduction in any medium, provided the original author(s) and source are credited.

## Electronic supplementary material

Supplementary material, approximately 4.09 MB.
