# Supplementary material for: Five new 5,6-seco-tremulane sesquiterpenoids from the basidiomycete Conocybe siliginea
Source: Nat Prod Bioprospect. 2013 Mar 21;3(2):48–51. doi: 10.1007/s13659-013-0003-1 (PMC4131662; doi:10.1007/s13659-013-0003-1)

Electronic Supplementary Material

Five new 5,6-*seco*-tremulane sesquiterpenoids from the basidiomycete *Conocybe siliginea*

Xiao-Yan YANG,[a,b] Tao FENG,[a] Jian-Hai DING,[a,b] Xia YIN,[a,b] Hua GUO,[a,b] Zheng-Hui LI,[a] and Ji-Kai LIU[a,]*

[a]State Key Laboratory of Phytochemistry and Plant Resources in West China, Kunming Institute of Botany, Chinese Academy of Sciences, Kunming 650201, China

[b]University of Chinese Academy of Sciences, Beijing 100049, China

Received 6 January 2013; Accepted 13 March 2013

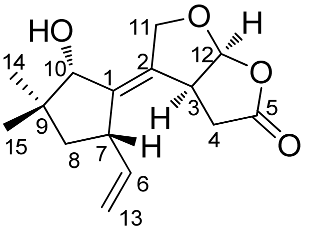

1

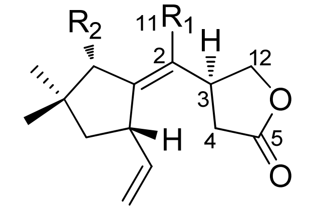

2 R_1 = CH_2OAc, R_2 = H
3 R_1 = CHO, R_2 = H
4 R_1 = CH_2OAc, R_2 = OH

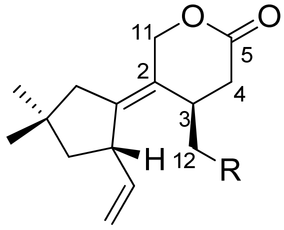

5 R = OAc
7 R = OH

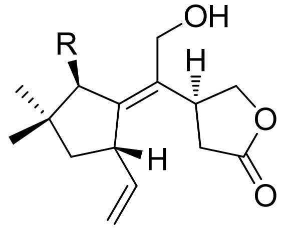

6 R = H
8 R = OH

Structures of compounds **1**–**8**

*To whom correspondence should be addressed. E-mail: jkliu@mail.kib.ac.cn

Figure S1–S7. NMR and MS of compound **1**.

Figure S8–S14. NMR and MS of compound **2**.

Figure S15–S21. NMR and MS of compound **3**.

Figure S22–S28. NMR and MS of compound **4**.

Figure S29–S35. NMR and MS of compound **5**.

Figure S1. ^{1}H NMR(400 MHz) spectrum of compound **1** in $CDCl_3$.

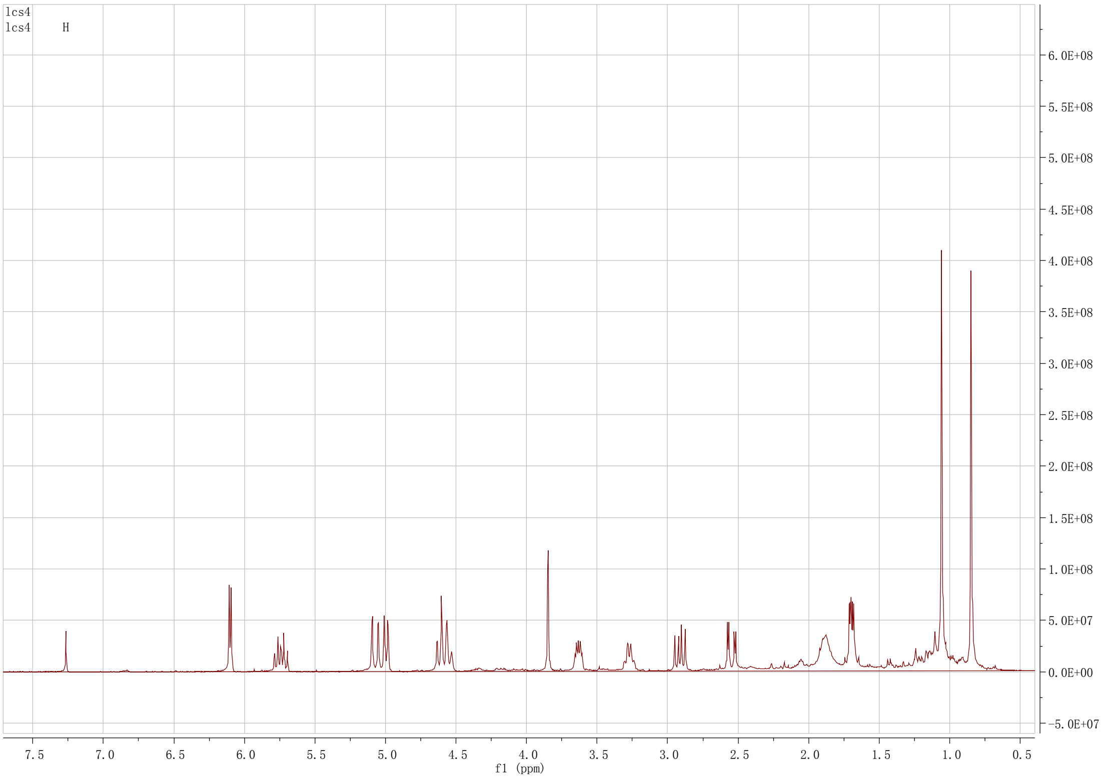

Figure S2. ^{13}C NMR(100 MHz) spectrum of compound **1** in $CDCl_3$.

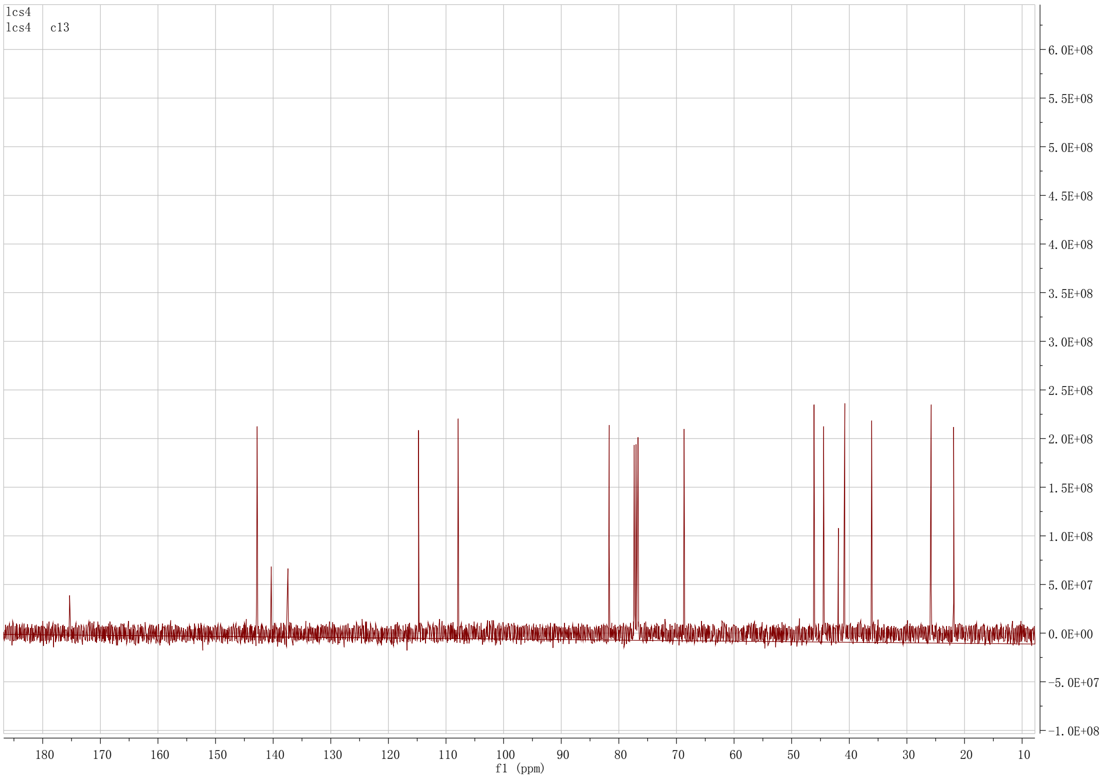

Figure S3. HSQC (500 MHz) spectrum of compound **1** in $CDCl_3$.

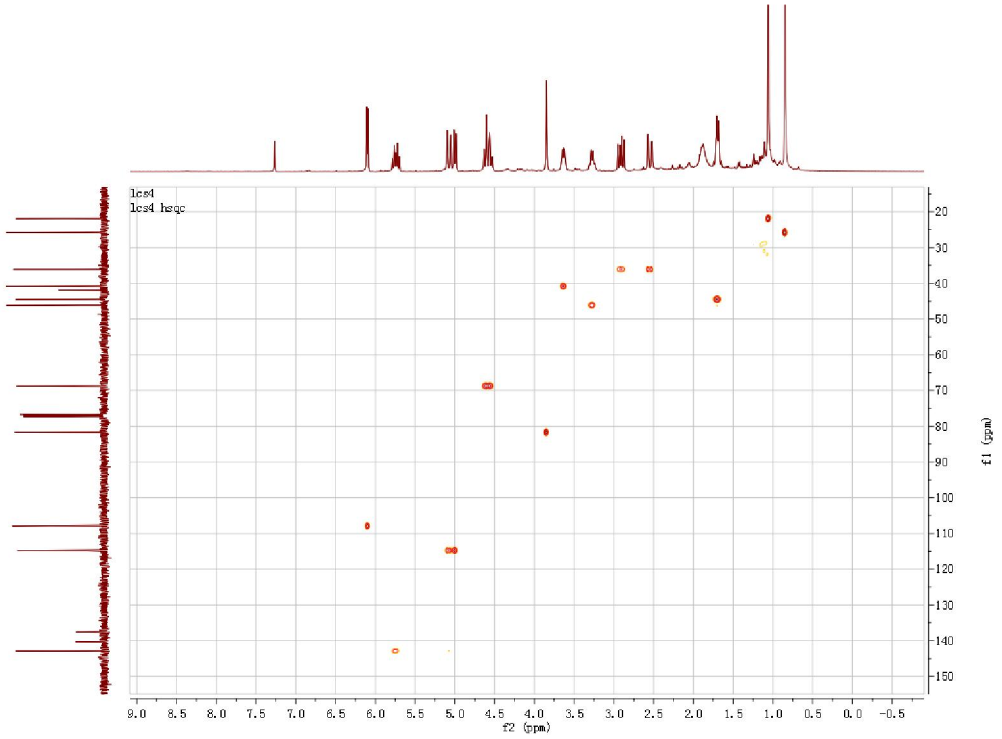

Figure S4. HMBC (500 MHz) spectrum of compound 1 in $CDCl_3$.

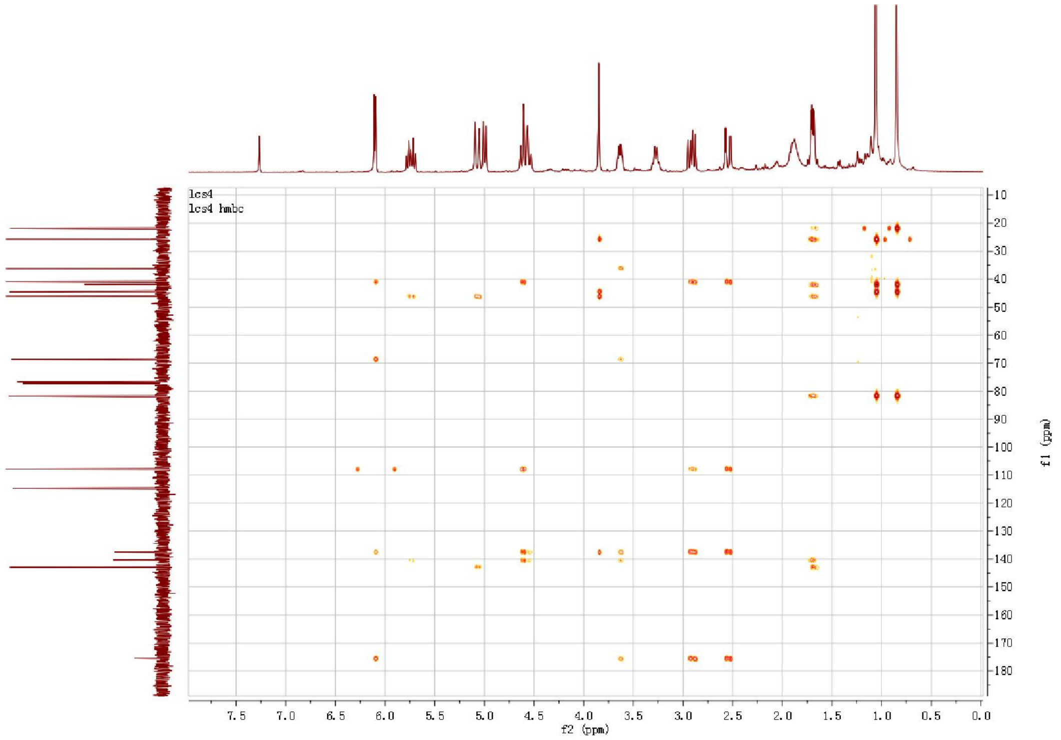

Figure S5. ^{1}H-^{1}H COSY (500 MHz) spectrum of compound **2** in $CDCl_3$.

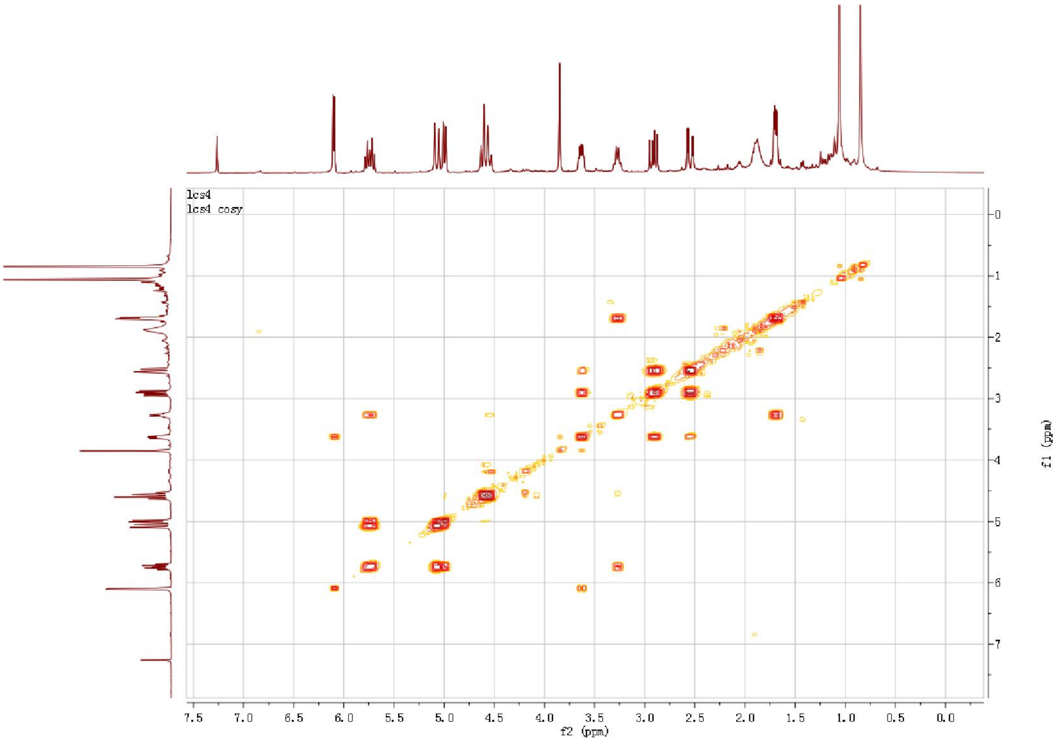

Figure S6. ROESY (500 MHz) spectrum of compound **1** in $CDCl_3$.

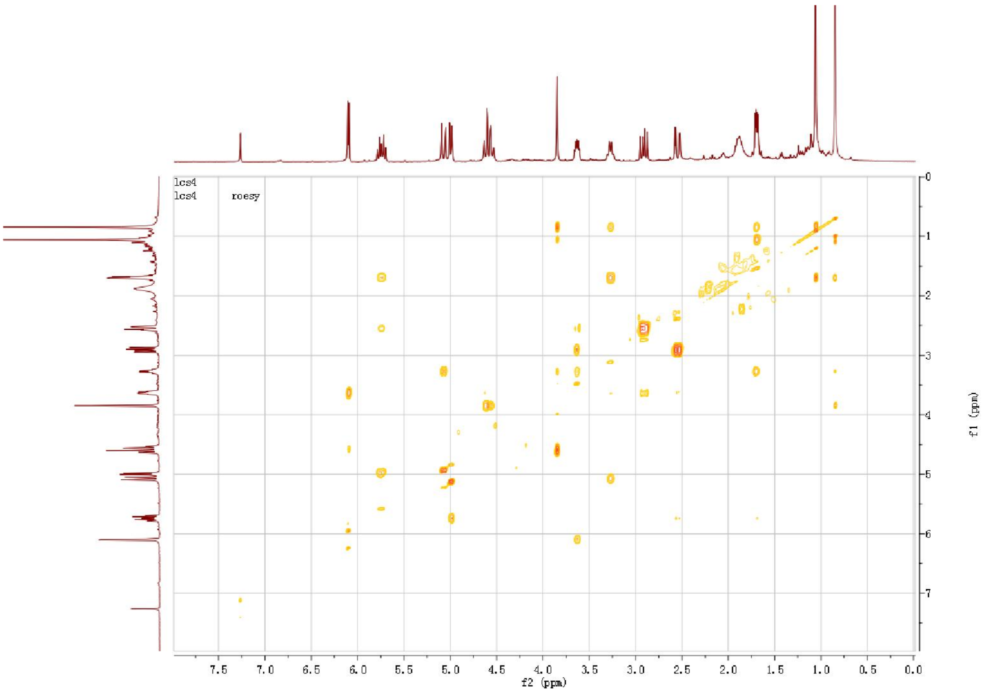

Figure S7. (+) HREIMS spectrum of compound **1**.

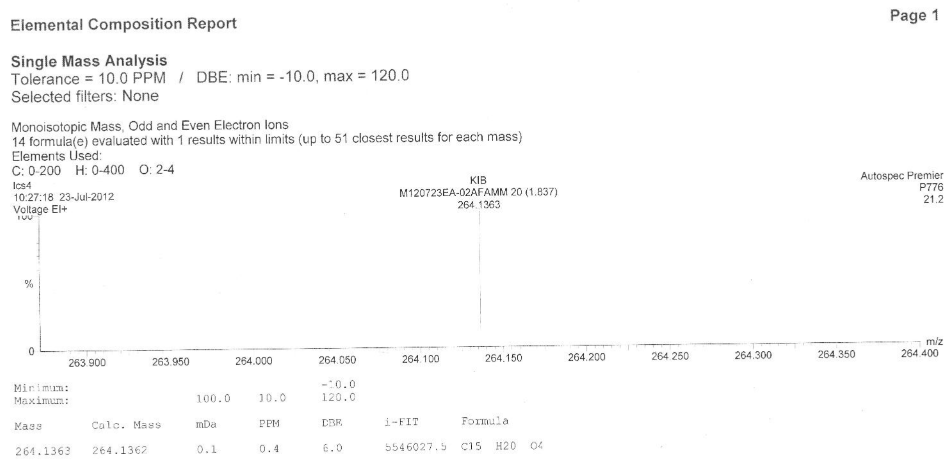

Figure S8. ^{1}H NMR(400 MHz) spectrum of compound **2** in $CDCl_3$.

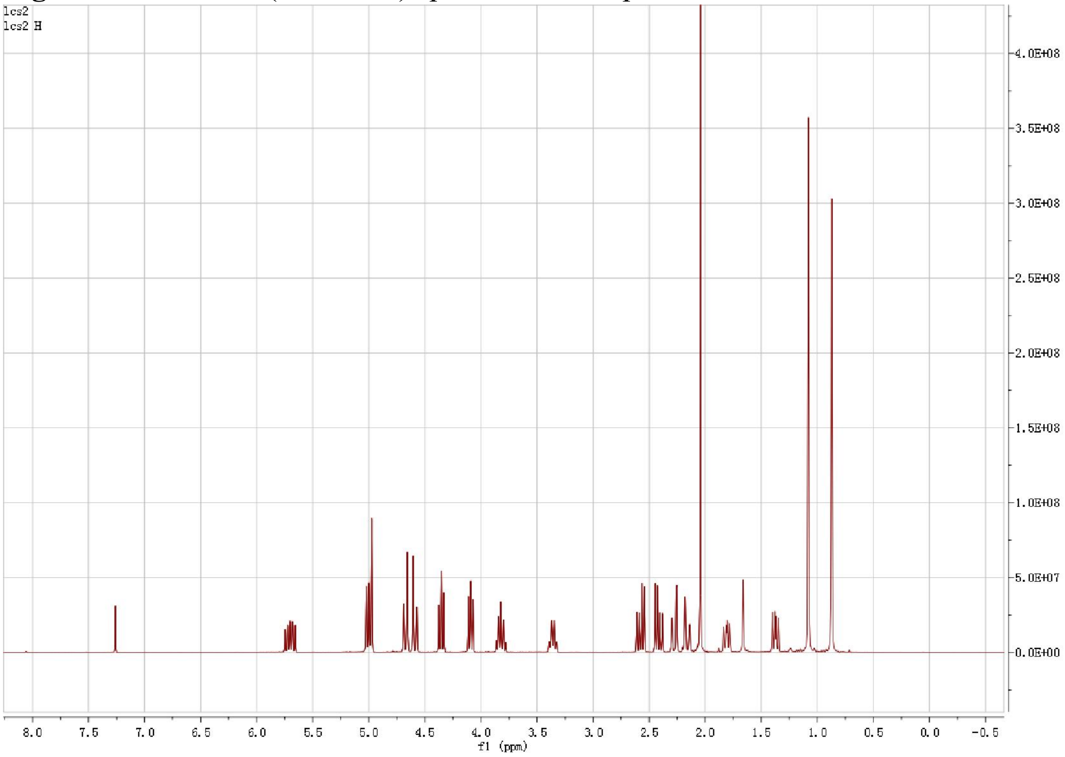

Figure S9. ^{13}C NMR(100 MHz) spectrum of compound **2** in $CDCl_3$.

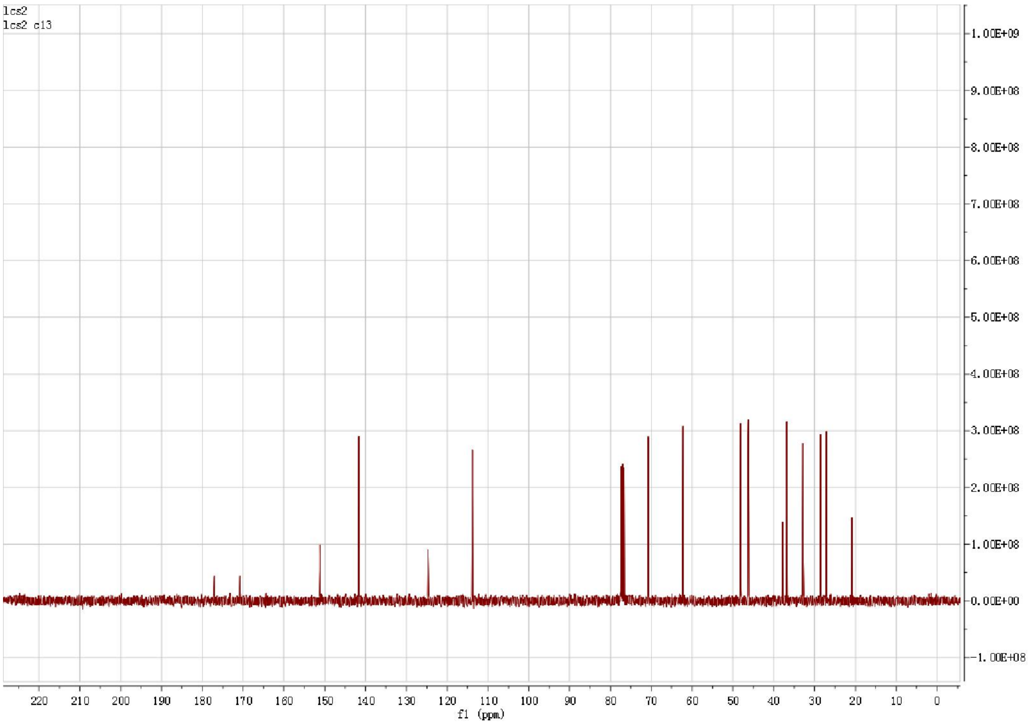

Figure S10. HSQC (500 MHz) spectrum of compound **2** in $CDCl_3$.

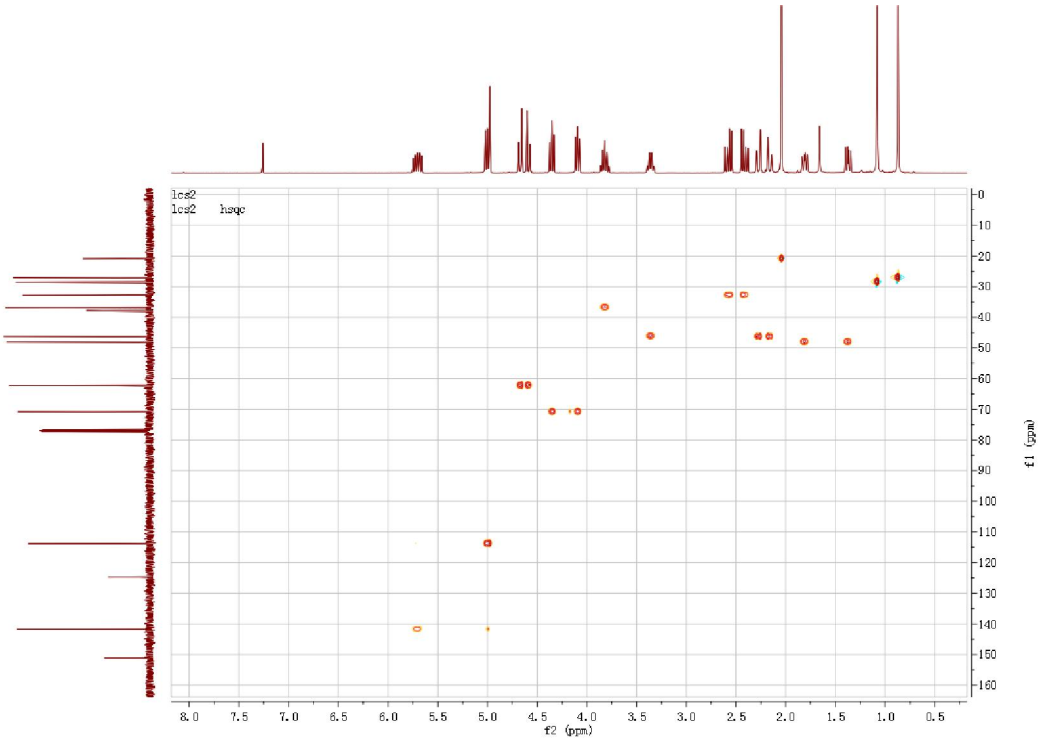

Figure S11. HMBC (500 MHz) spectrum of compound **2** in $CDCl_3$.

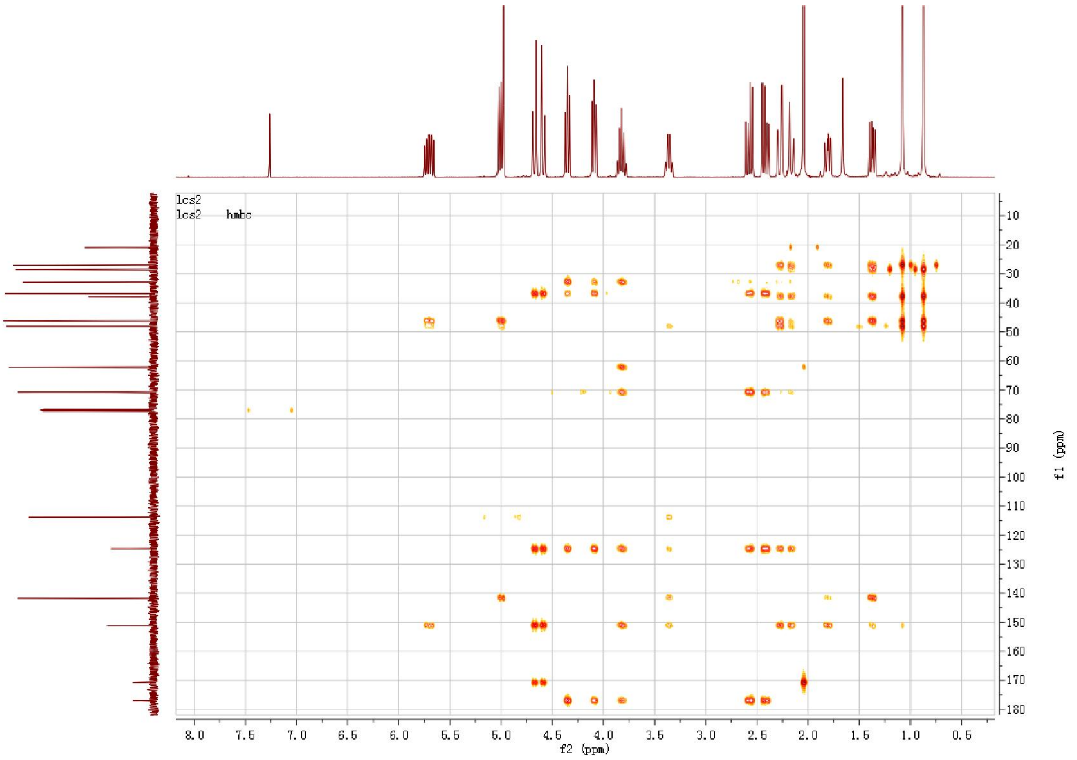

Figure S12. ^{1}H-^{1}H COSY (500 MHz) spectrum of compound **2** in $CDCl_3$.

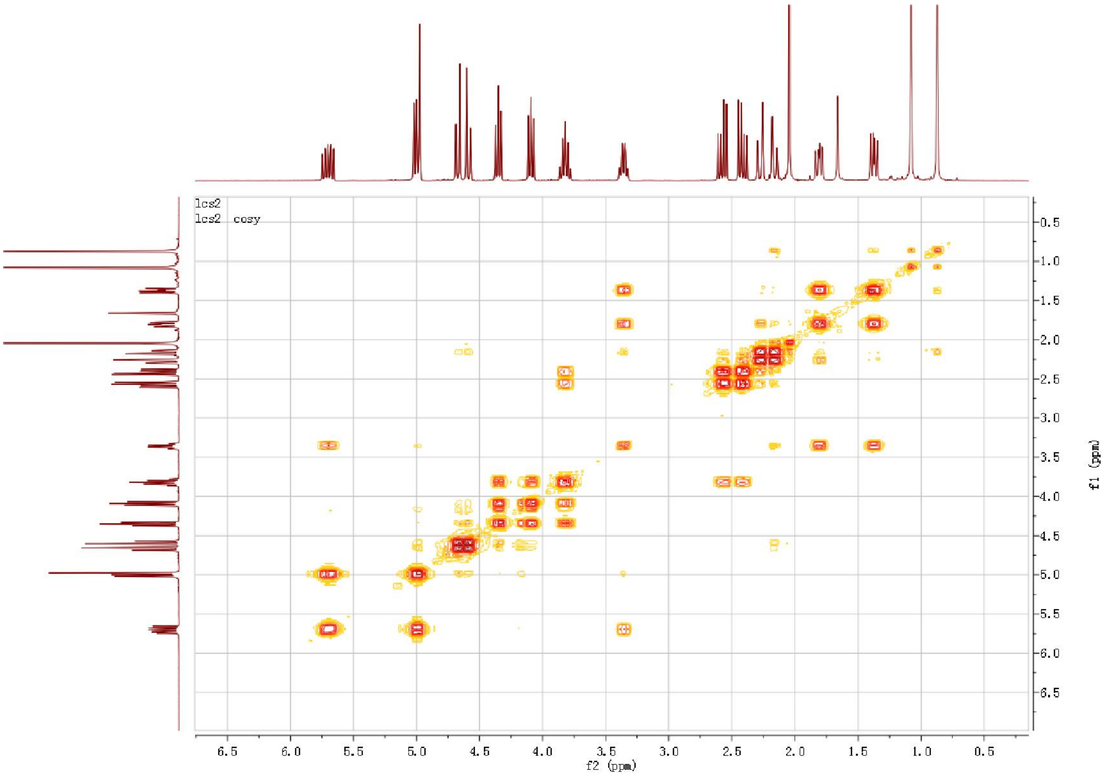

Figure S13. ROESY (500 MHz) spectrum of compound **2** in $CDCl_3$.

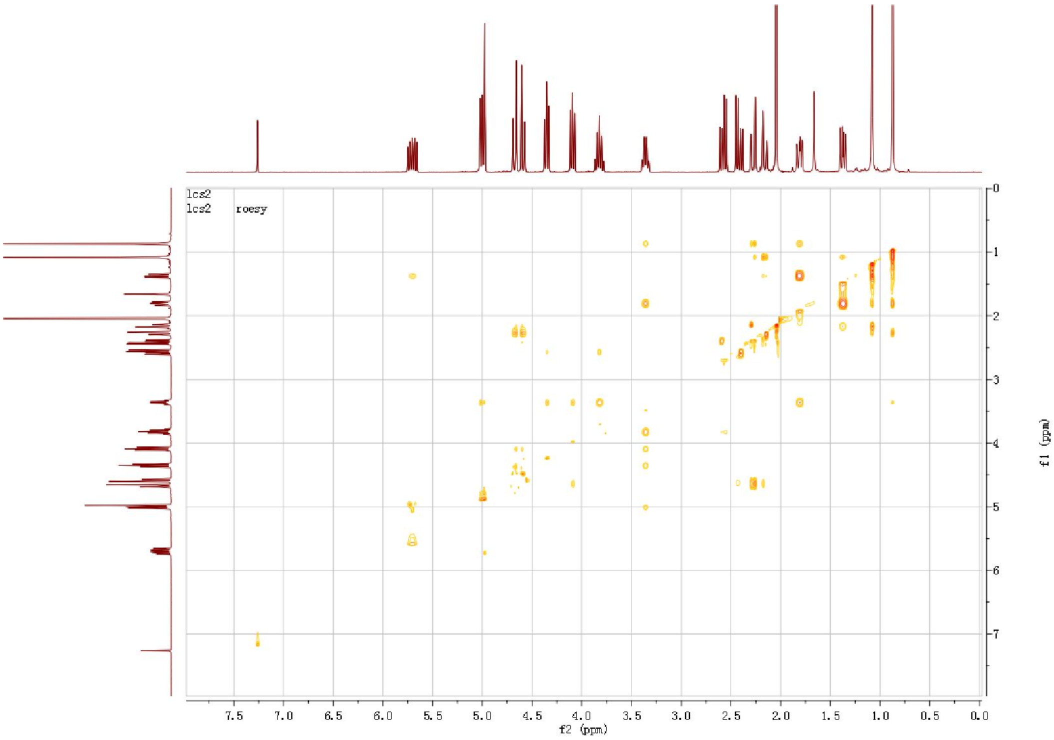

Figure S14. (+) HRESIMS spectrum of compound **2**.

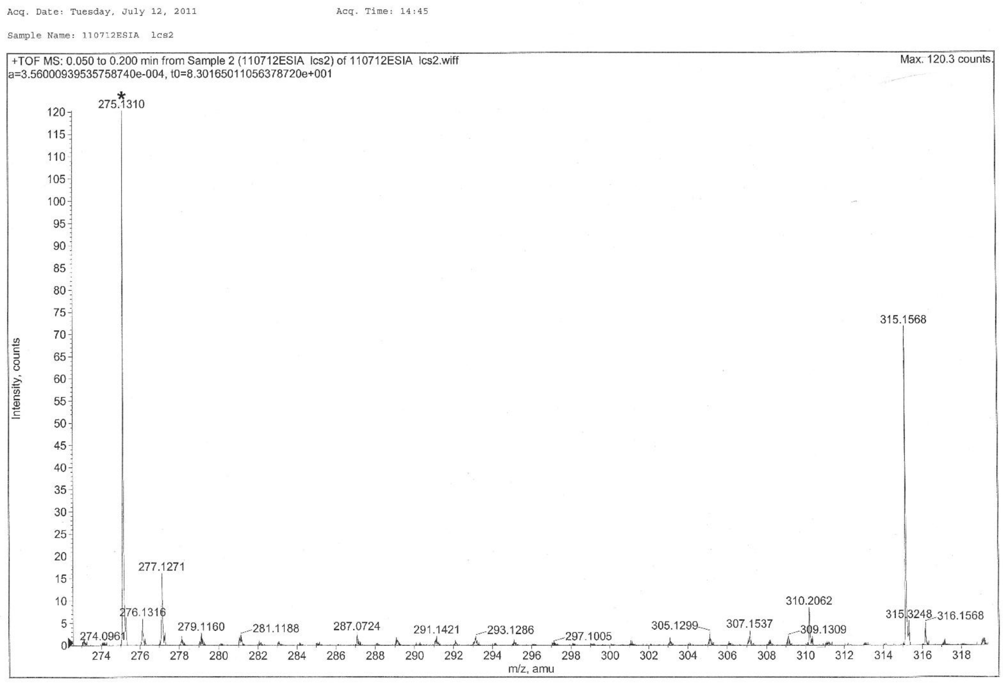

Figure S15. ^{1}H NMR (400 MHz) spectrum of compound **3** in $CDCl_3$.

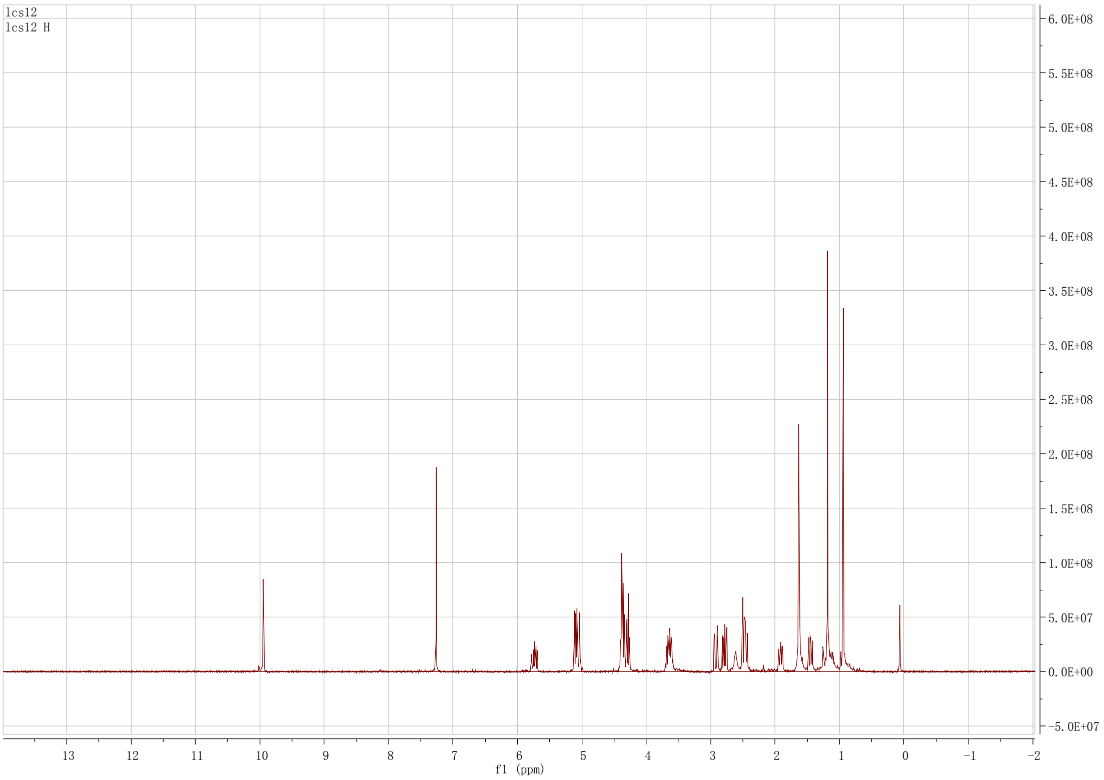

Figure S16. ^{13}C NMR (100 MHz) spectrum of compound **3** in $CDCl_3$.

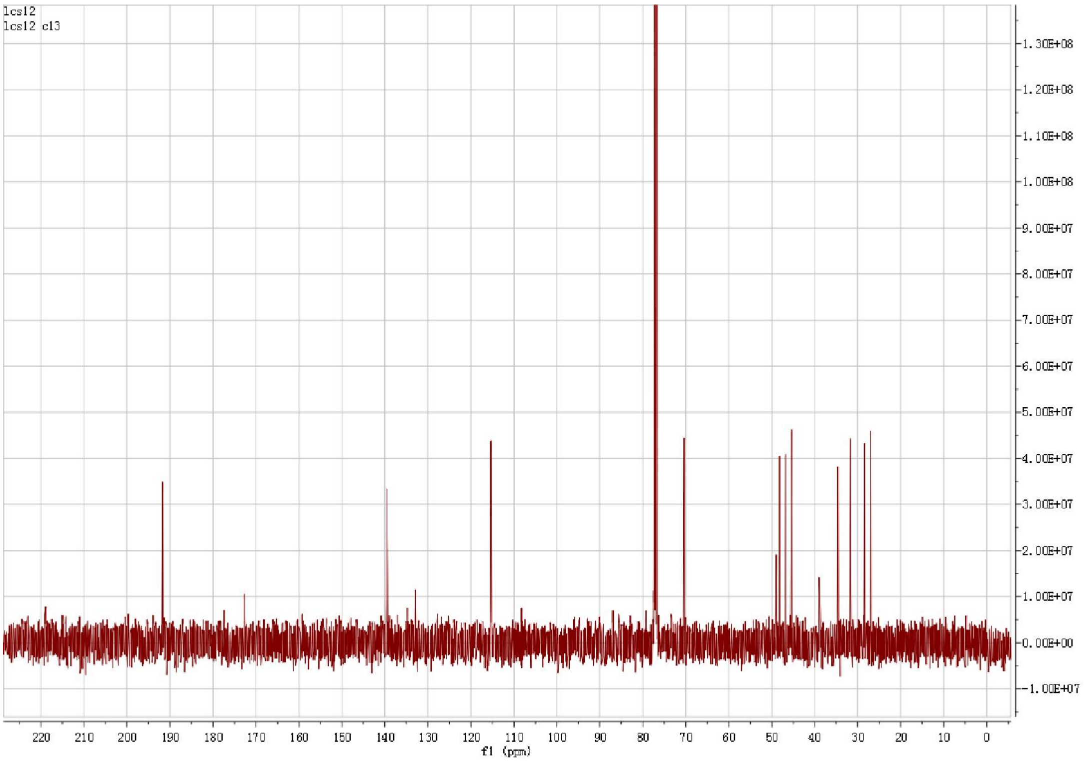

Figure S17. HSQC (500 MHz) spectrum of compound **3** in $CDCl_3$.

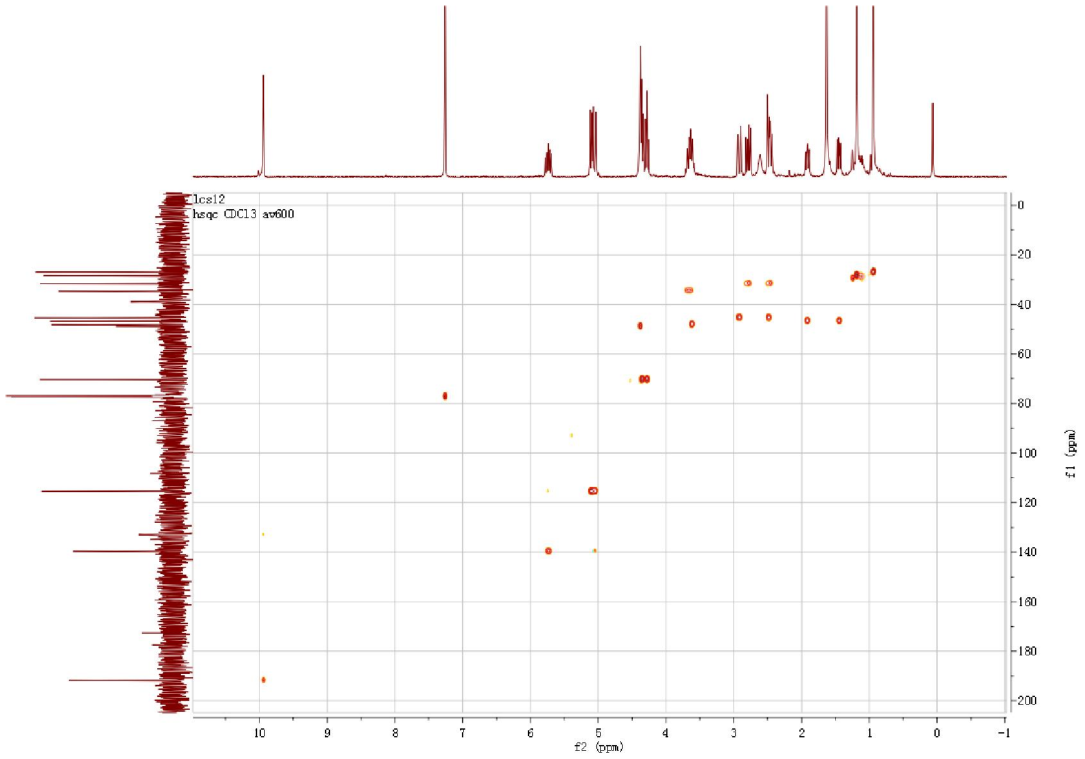

Figure S18. HMBC (500 MHz) spectrum of compound **3** in $CDCl_3$.

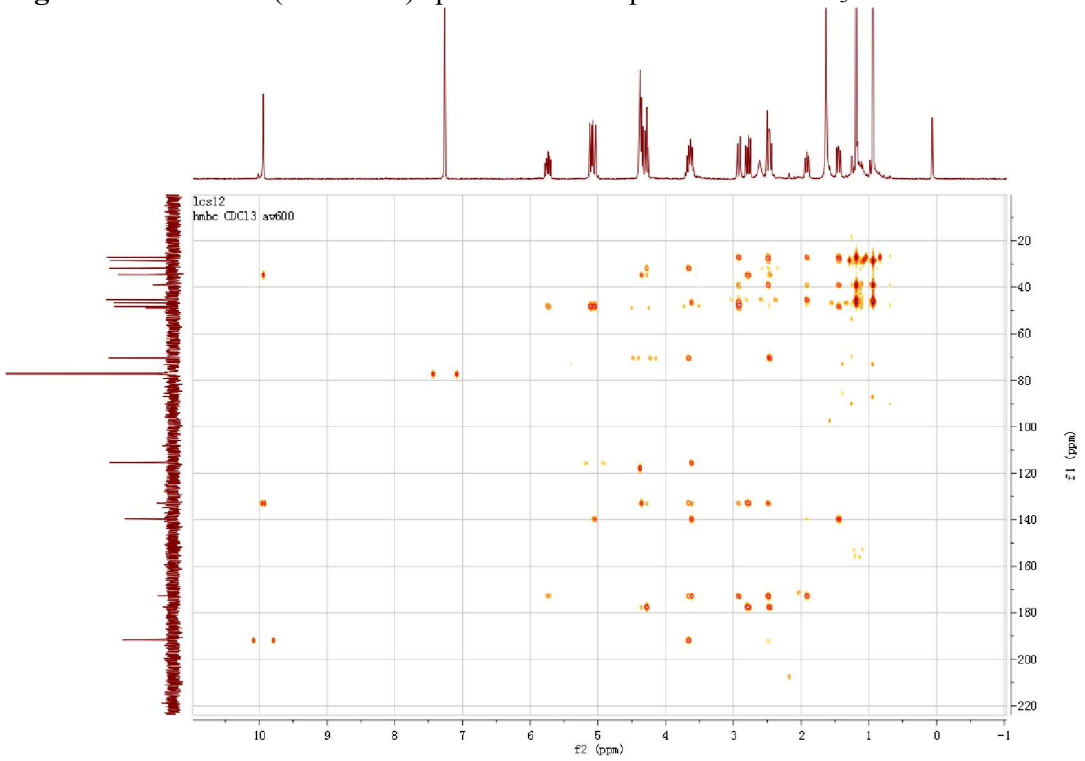

Figure S19. ^{1}H-^{1}H COSY (600 MHz) spectrum of compound **3** in $CDCl_3$.

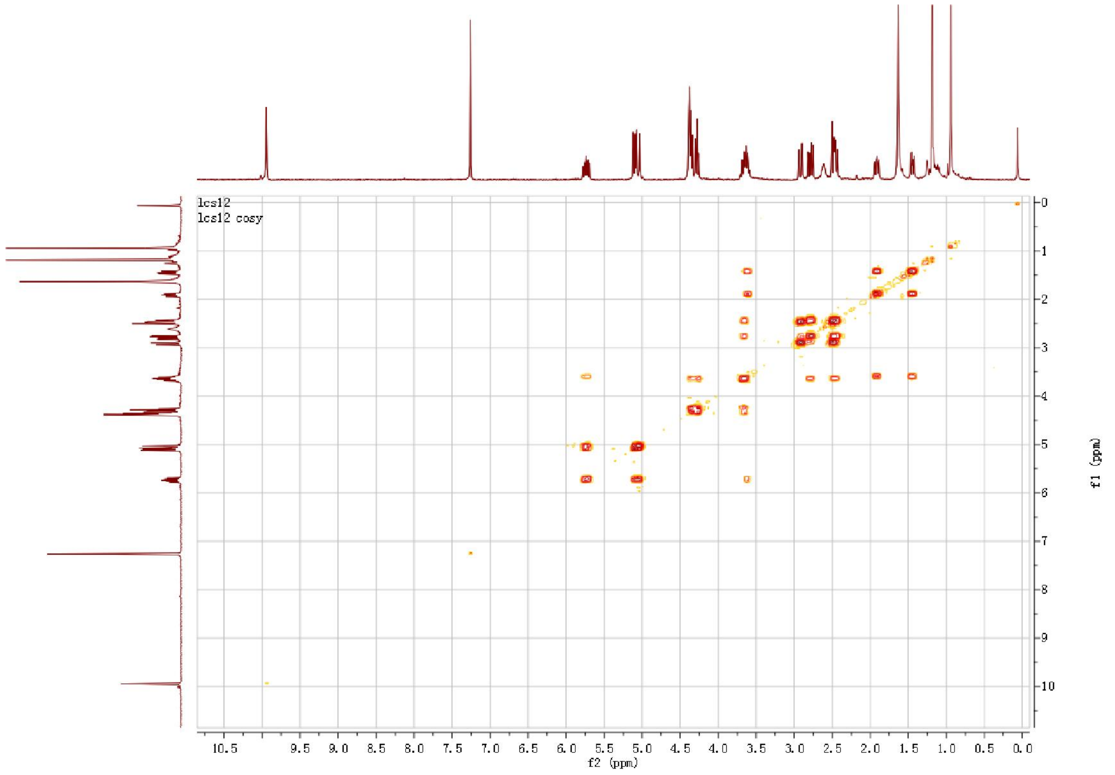

Figure S20. ROESY (600 MHz) spectrum of compound **3** in $CDCl_3$.

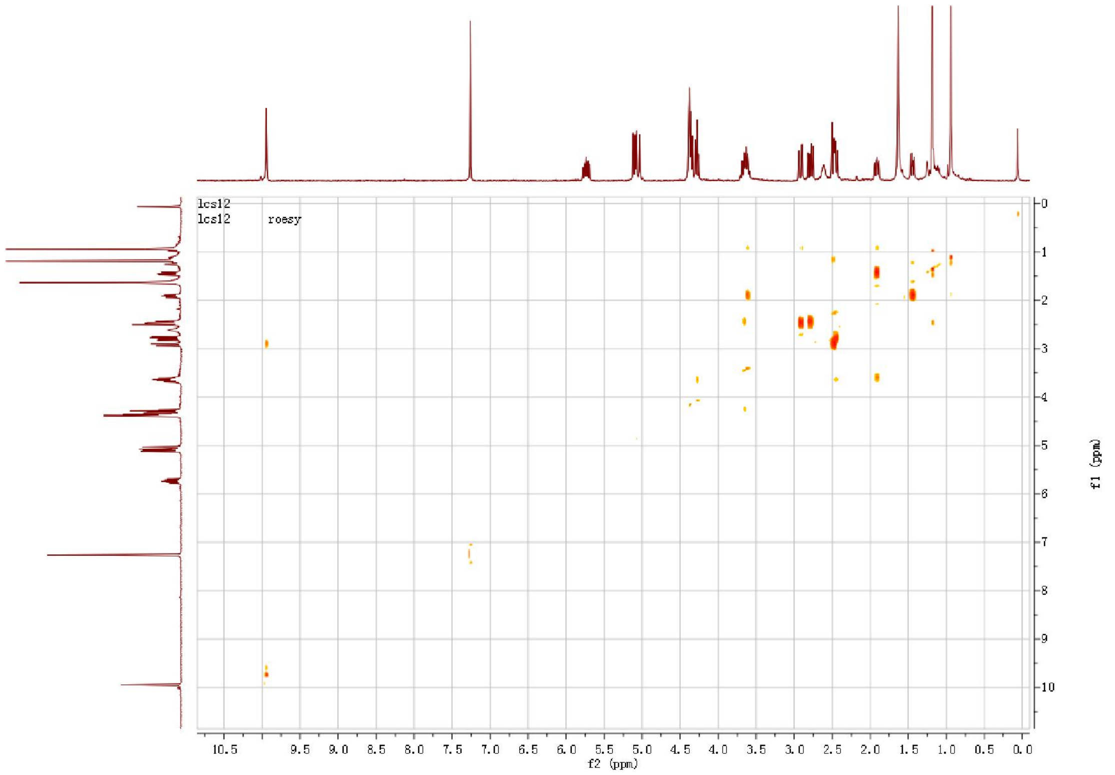

Figure S21. (+) HRESIMS spectrum of compound **3**.

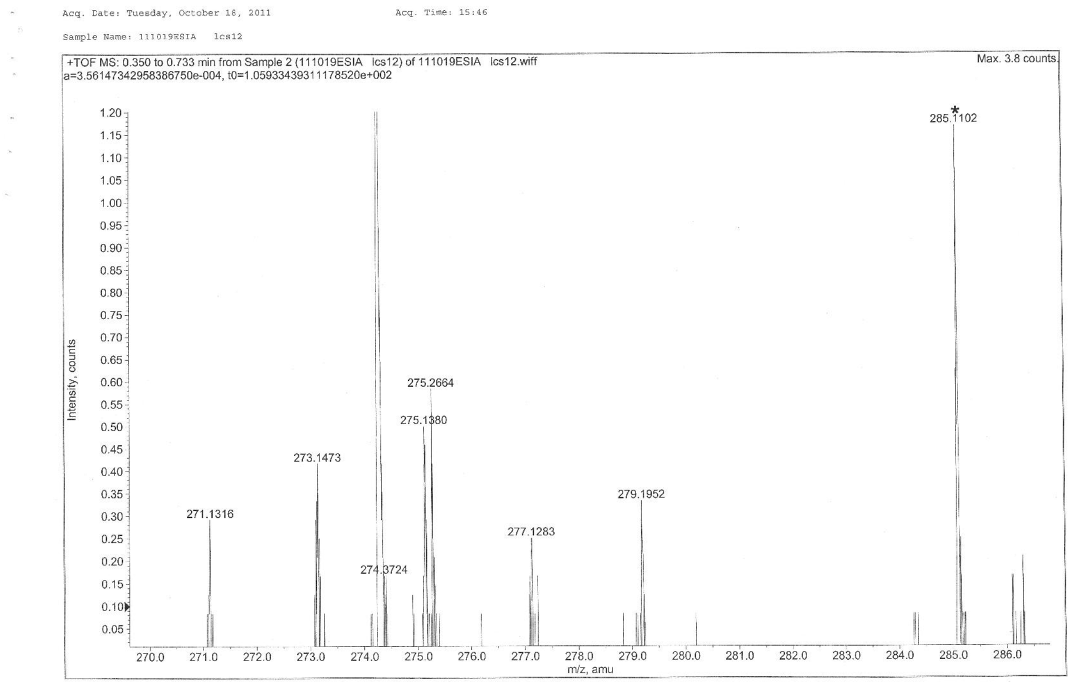

Figure S22. ^{1}H NMR (400 MHz) spectrum of compound **4** in $CDCl_3$.

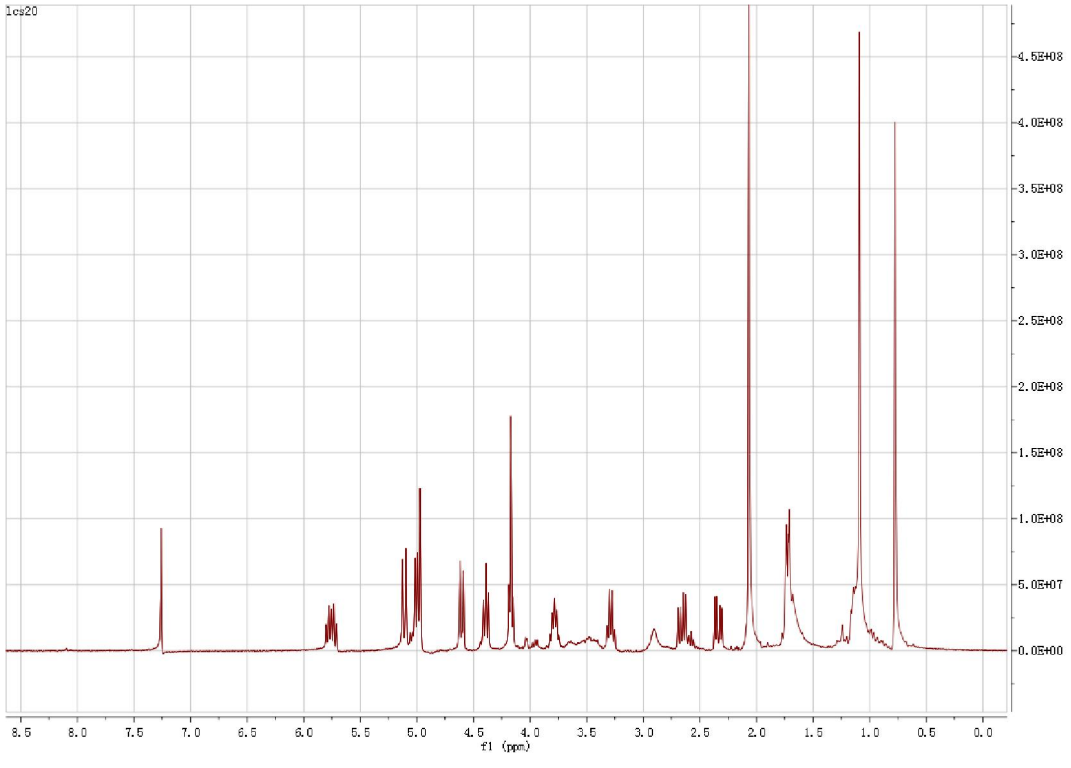

Figure S23. ^{13}C NMR (100 MHz) spectrum of compound **4** in $CDCl_3$.

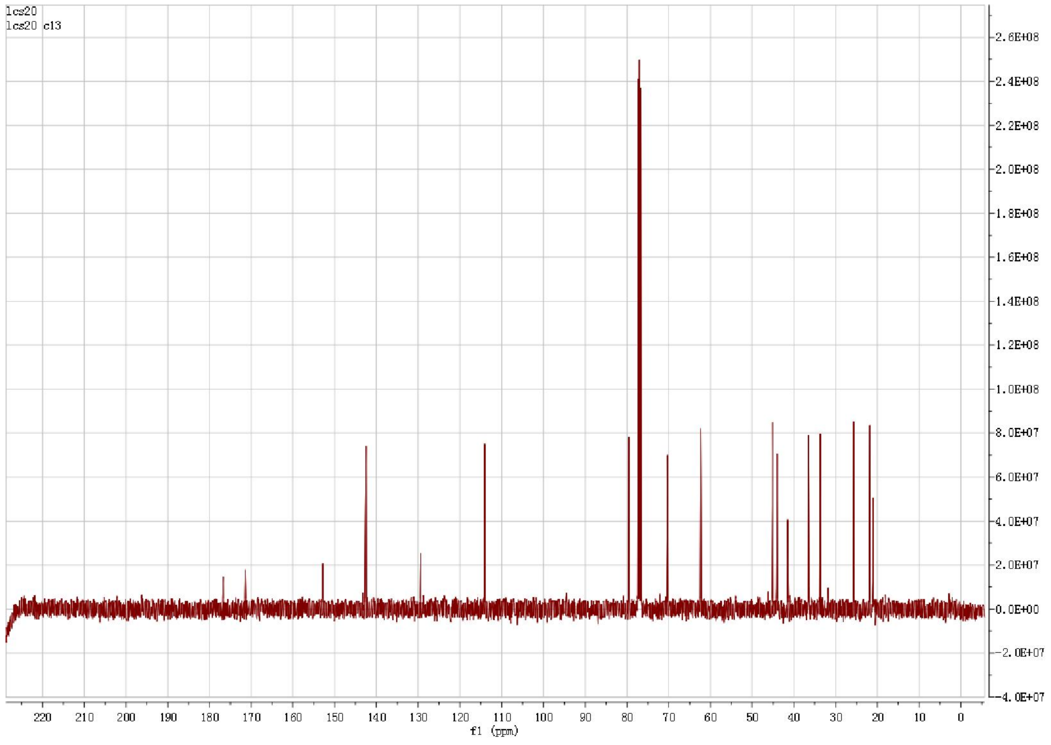

Figure S24. HSQC (500 MHz) spectrum of compound **4** in $CDCl_3$.

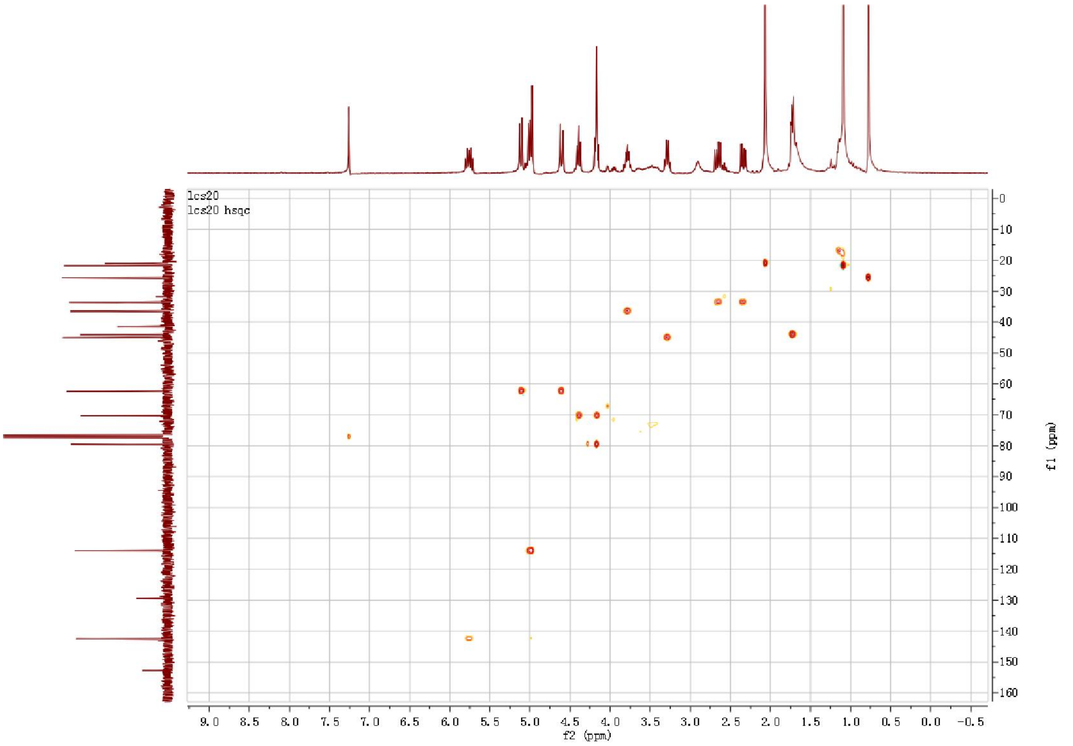

Figure S25. HMBC (500 MHz) spectrum of compound **4** in $CDCl_3$.

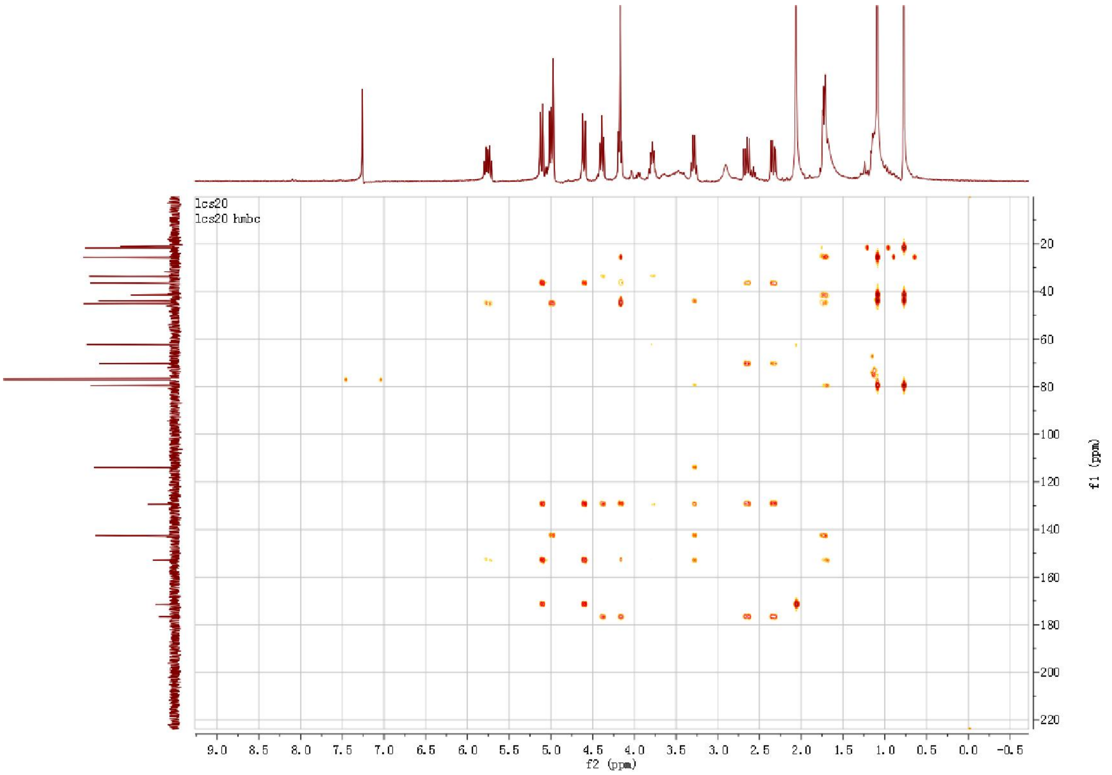

Figure S26. ^{1}H-^{1}H COSY (500 MHz) spectrum of compound **4** in $CDCl_3$.

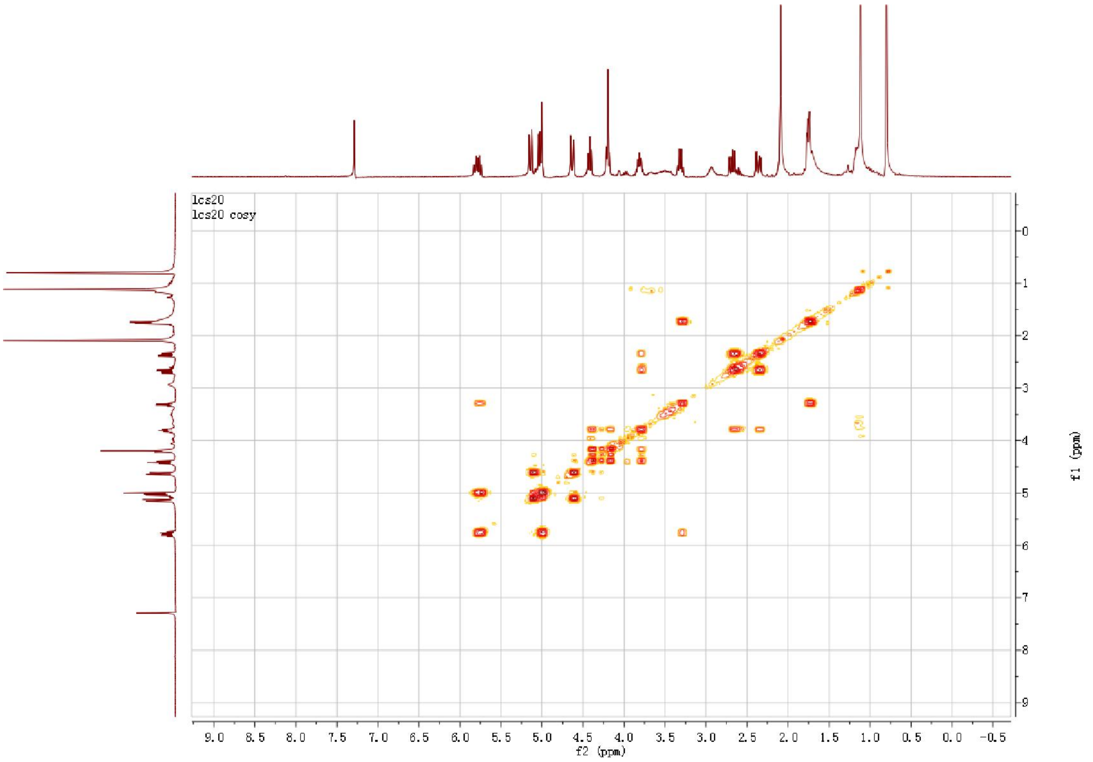

Figure S27. ROESY (500 MHz) spectrum of compound **4** in $CDCl_3$.

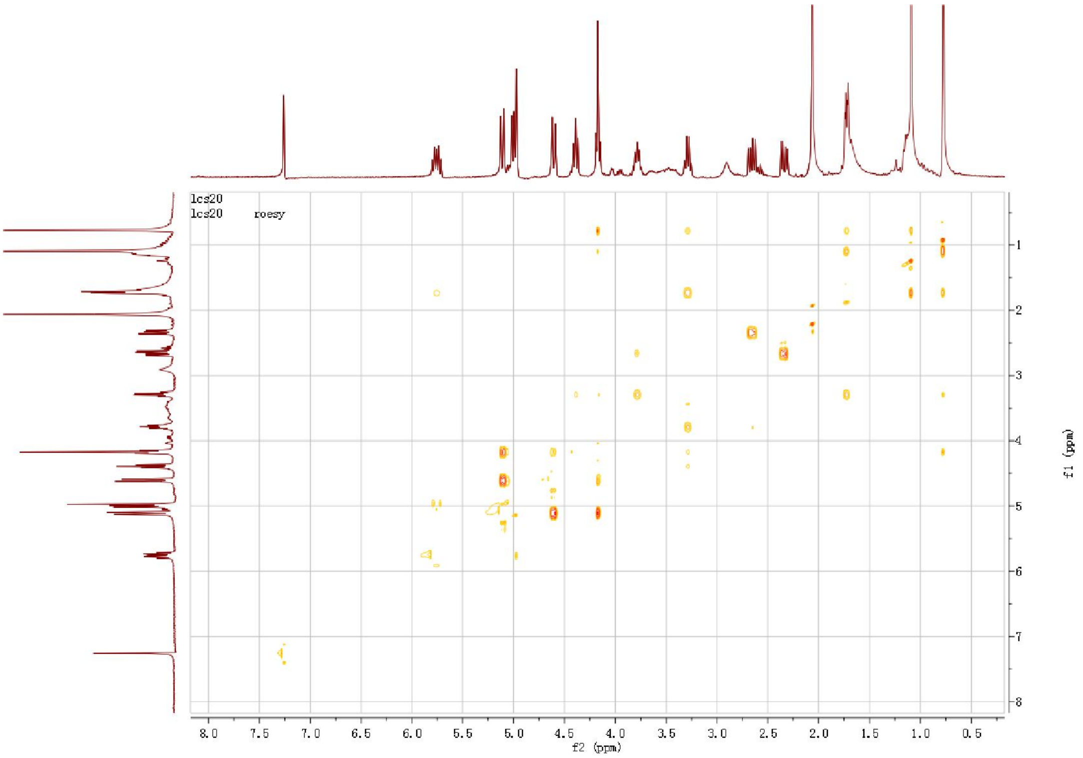

Figure S28. (+) HRESIMS spectrum of compound **4**.

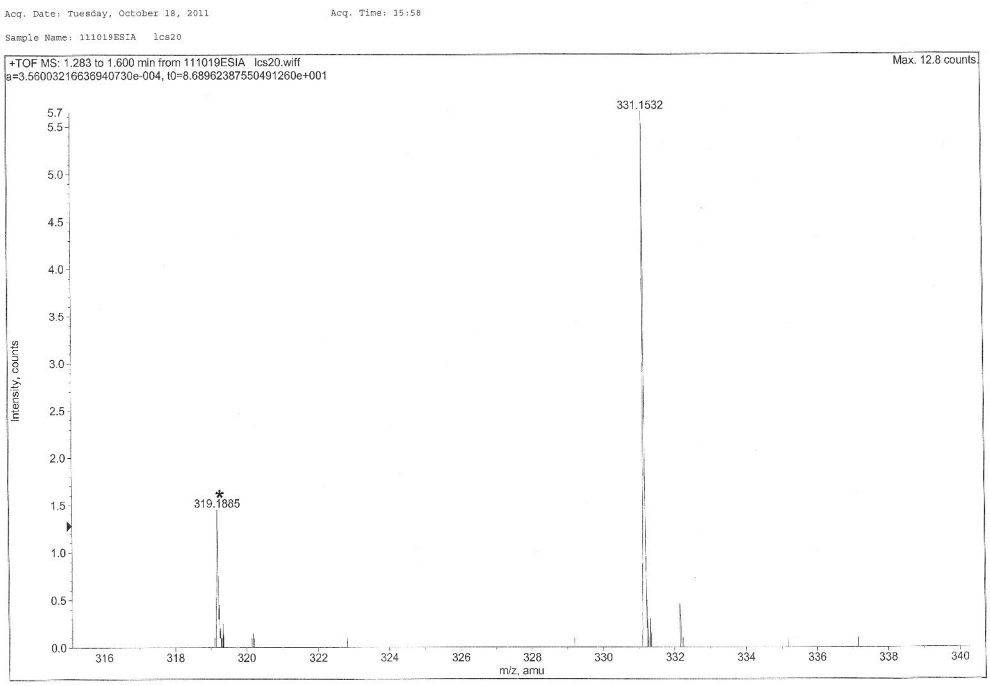

Figure S29. ^{1}H NMR (400 MHz) spectrum of compound **5** in $CDCl_3$.

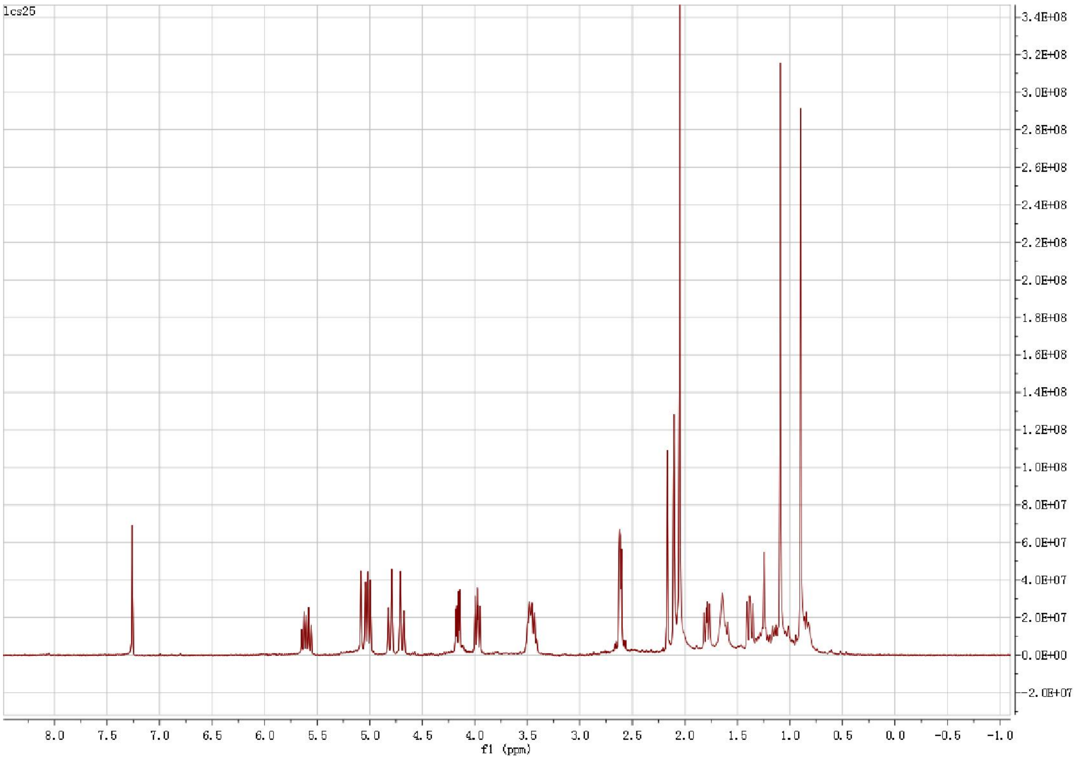

Figure S30. ^{13}C NMR (100 MHz) spectrum of compound **5** in $CDCl_3$.

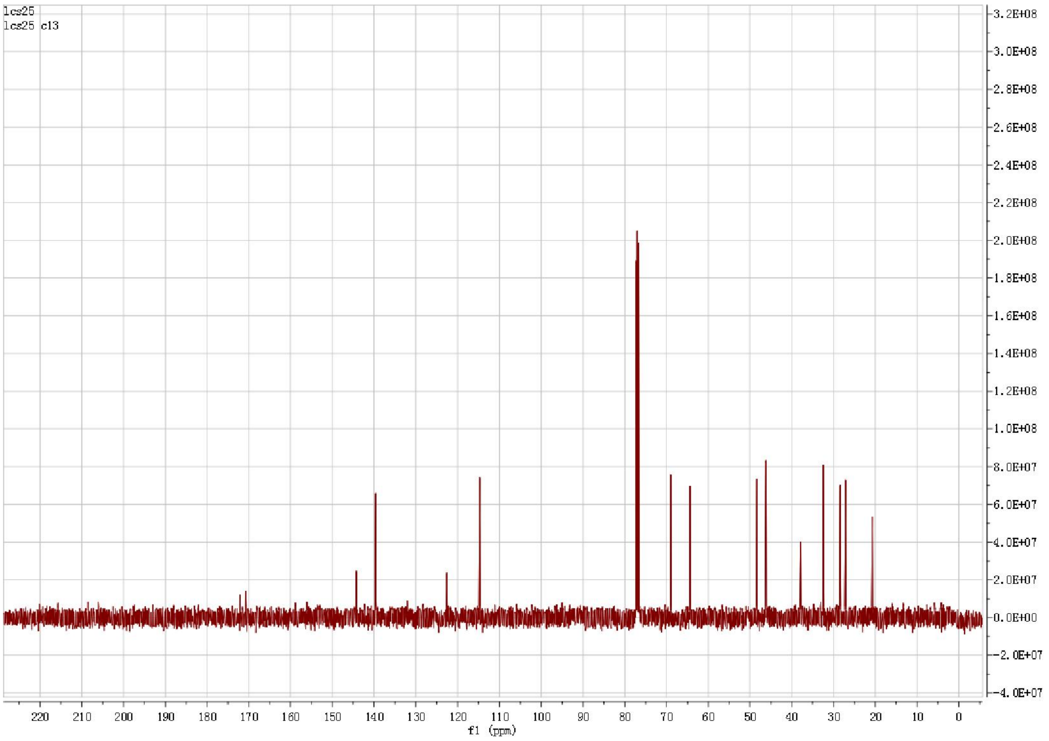

Figure S31. HSQC (500 MHz) spectrum of compound **5** in $CDCl_3$.

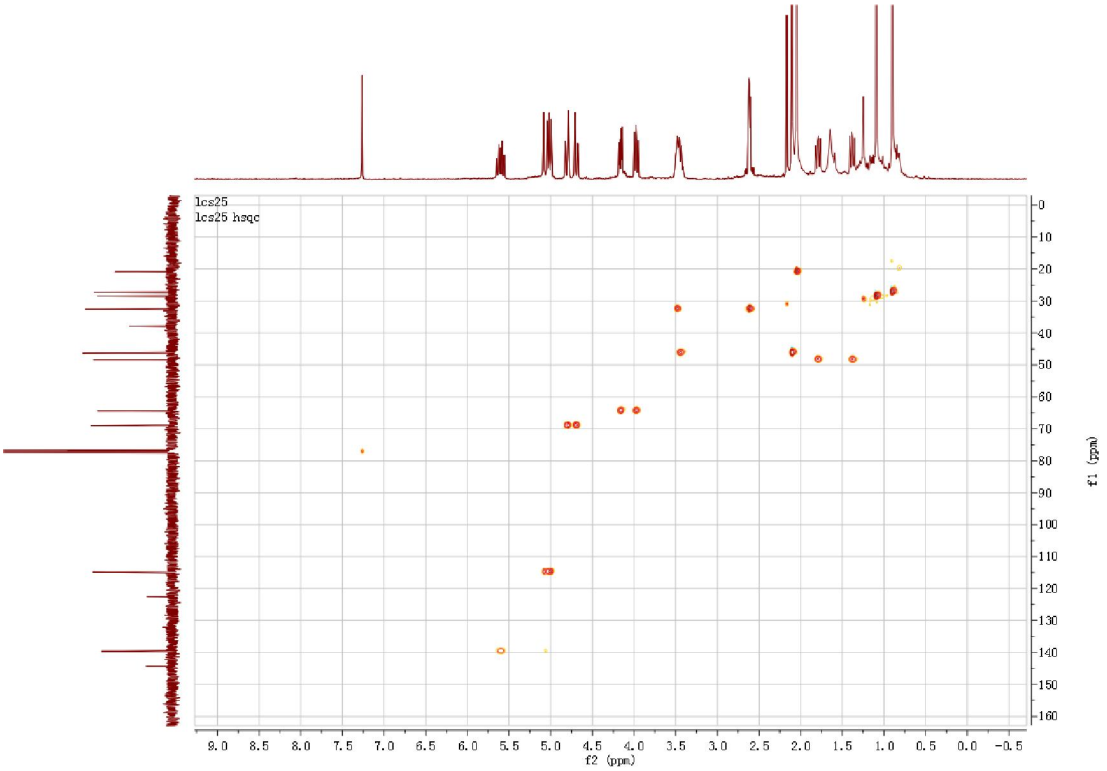

Figure S32. HMBC (500 MHz) spectrum of compound **5** in $CDCl_3$.

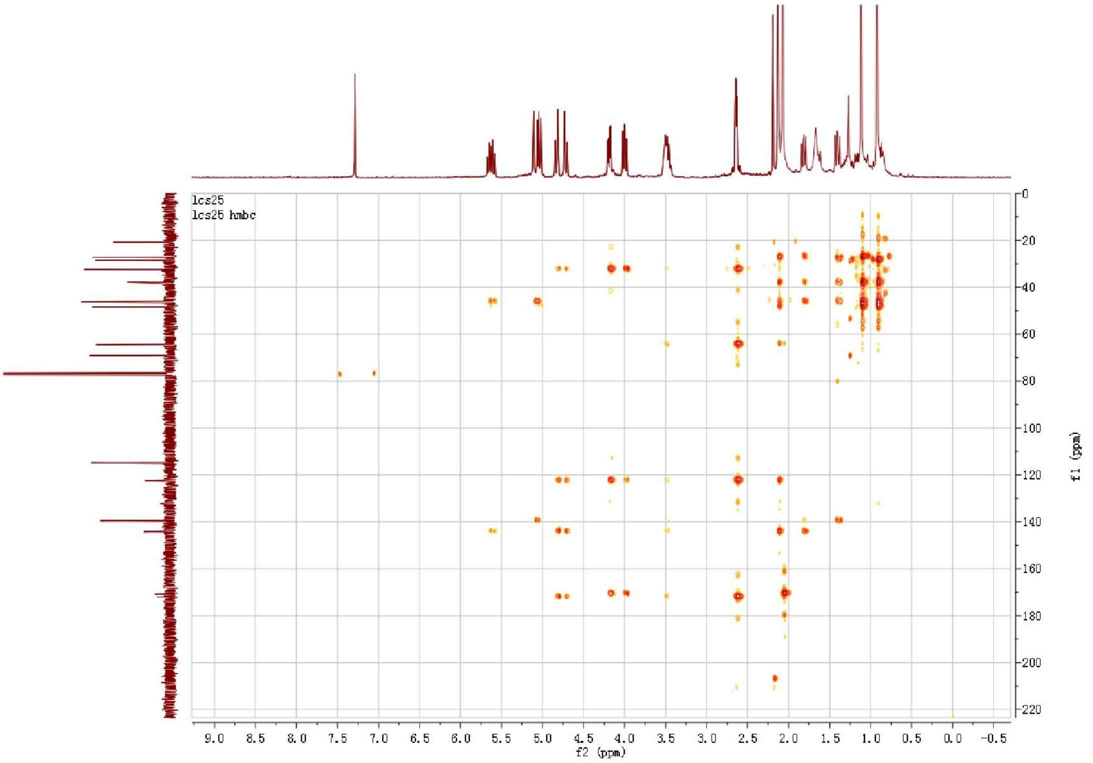

Figure S33. ^{1}H-^{1}H COSY (500 MHz) spectrum of compound **5** in $CDCl_3$.

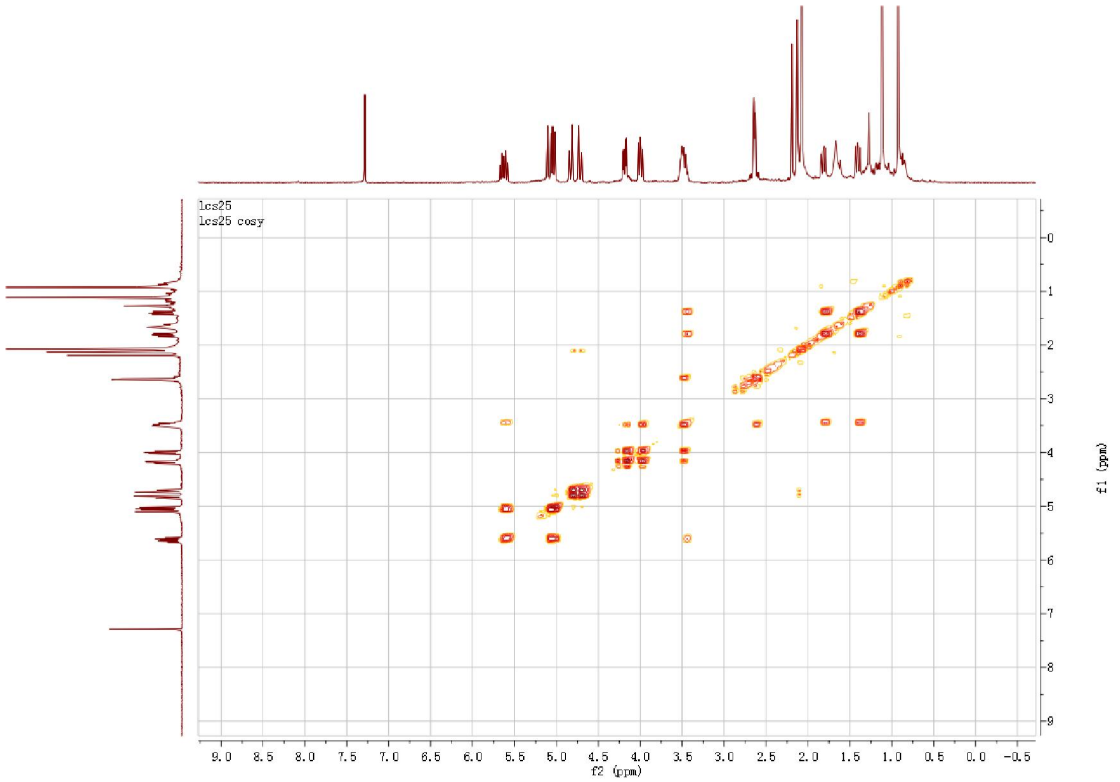

Figure S34. ROESY (500 MHz) spectrum of compound **5** in $CDCl_3$.

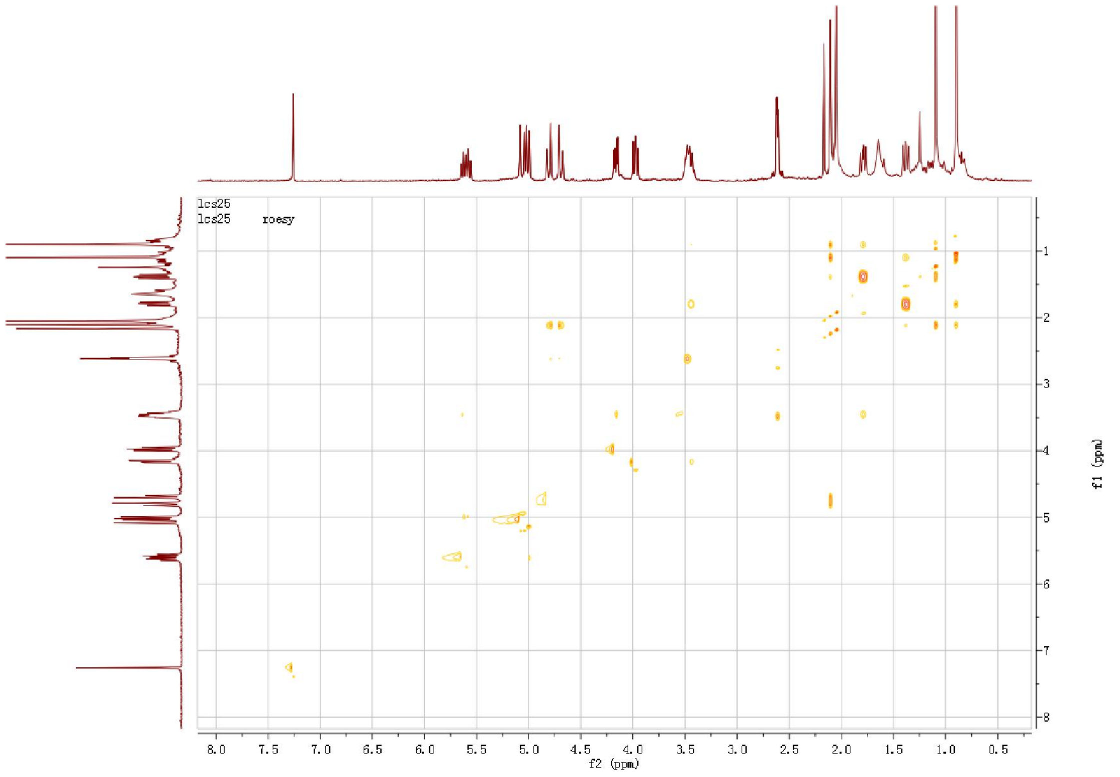

Figure S35. (+) HRESIMS spectrum of compound **5**.

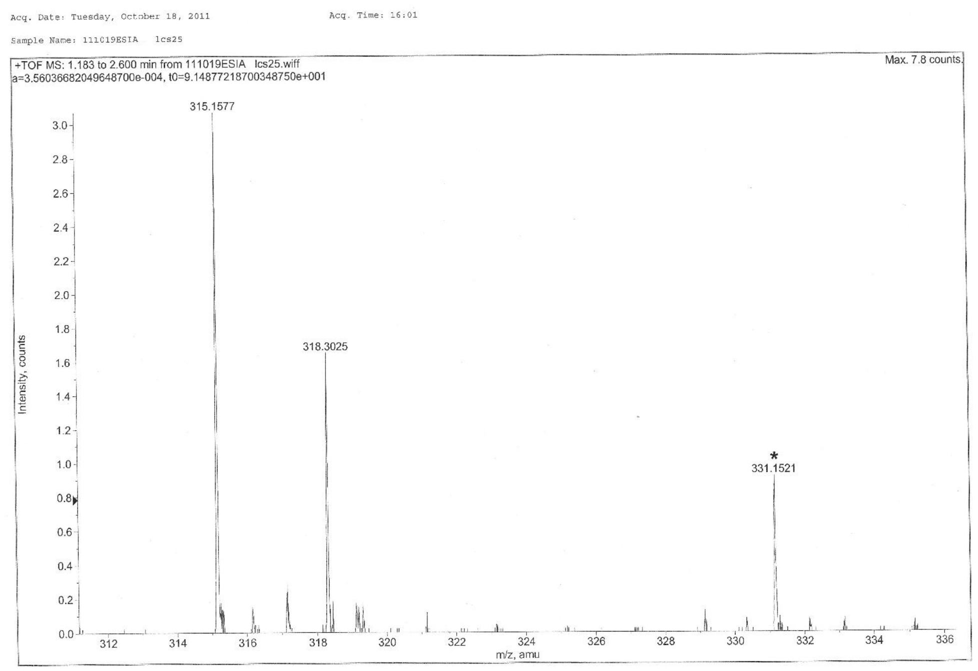

Supplement: Supplementary file 1 — Supplementary material, approximately 4.09 MB. [file 13659_2013_3_MOESM1_ESM.pdf]
